# Supplementary material for: Protocol for the evaluation of a pilot implementation of essential interventions for the prevention of cardiovascular diseases in primary healthcare in the Republic of Moldova
Source: BMJ Open. 2019 Jul 4;9(7):e025705. doi: 10.1136/bmjopen-2018-025705 (PMC6615880; doi:10.1136/bmjopen-2018-025705)
Supplement: Author's manuscript [file bmjopen-2018-025705.draft_revisions.pdf]

BMJ Open is committed to open peer review. As part of this commitment we make the peer review history of every article we publish publicly available.

When an article is published we post the peer reviewers' comments and the authors' responses online. We also post the versions of the paper that were used during peer review. These are the versions that the peer review comments apply to.

The versions of the paper that follow are the versions that were submitted during the peer review process. They are not the versions of record or the final published versions. They should not be cited or distributed as the published version of this manuscript.

BMJ Open is an open access journal and the full, final, typeset and author-corrected version of record of the manuscript is available on our site with no access controls, subscription charges or pay-per-view fees (http://bmjopen.bmj.com).

If you have any questions on BMJ Open's open peer review process please email info.bmjopen@bmj.com

BMJ Open

Protocol for the Evaluation of a Pilot Implementation of Essential Interventions for the Prevention of Cardiovascular Diseases in Primary Health Care in the Republic of Moldova

Journal:	*BMJ Open*
Manuscript ID	bmjopen-2018-025705
Article Type:	Protocol
Date Submitted by the Author:	01-Aug-2018
Complete List of Authors:	Collins, Dylan; University of British Columbia,<br>Ciobanu, Angela<br>Laatikainen, Tiina; National Institute for Health and Welfare, Epidemiology and Health Promotion<br>Curocichin, Ghenadie<br>Salaru, Virginia<br>Zatic, Tatiana<br>Anisei, Angela<br>Farrington, Jill
Keywords:	Cardiology < INTERNAL MEDICINE, PRIMARY CARE, Quality in health care < HEALTH SERVICES ADMINISTRATION & MANAGEMENT, Protocols & guidelines < HEALTH SERVICES ADMINISTRATION & MANAGEMENT

SCHOLARONE™
Manuscripts

Protocol for the Evaluation of a Pilot Implementation of Essential Interventions for the Prevention of Cardiovascular Diseases in Primary Health Care in the Republic of Moldova

Dylan R. J. Collins[1], Angela Ciobanu[2], Tiina Laatikainen[3], Ghenadie Curocichin[4], Virginia Salaru[4], Tatiana Zatic[5], Angela Anisei[6], Jill L. Farrington[7]

[1]University of British Columbia, Vancouver, Canada
[2]World Health Organization Country Office in Republic of Moldova, Chisinau, Republic of Moldova
[3]Institute of Public Health and Clinical Nutrition, Helsinki, Finland
[4]Family Medicine Department, Nicolae Testemitanu State Medical and Pharmaceutical University, Chisinau, Republic of Moldova
[5]Primary, Emergency and Community Health Policies Department, Ministry of Health, Labour and Social Protection, Chisinau, Republic of Moldova
[6]Department on Quality Management of Health Services, National Public Health Agency, Republic of Moldova
[7]World Health Organization Regional Office for Europe, Copenhagen, Denmark

Correspondence to Dr. Dylan Collins at dylan.collins@alumni.ubc.ca

For peer review only - http://bmjopen.bmj.com/site/about/guidelines.xhtml

ABSTRACT

Introduction

Nearly 90% of all deaths in Republic of Moldova are caused by NCDs, the majority of which (55%) are caused by CVD. In addition to reducing premature mortality from CVD, it is estimated that strengthening primary health care could cut the number hypertension-related hospital admissions and diabetes-related hospitalizations in half. The aim of this evaluation is to determine the feasibility of implementing and evaluating essential interventions for the prevention of CVD in primary health care in Republic of Moldova, with a view toward national scale-up.

Methods and Analysis

A national steering group including international experts will be convened to adapt WHO PEN protocols one and two to the health system of Republic of Moldova, develop and conduct training of primary health care workers, and test a core set of indicators to monitor the quality of care and change in clinical practice. To evaluate the impact of this pilot implementation, a pragmatic, sequential mixed methods explanatory design, composed of quantitative and qualitative strands of equal weight, will be used. Twenty primary health care centres will be selected and randomized to the training and implementation arm (n=10) and the usual care arm (n=10). At baseline and 12 months follow-up, a standardized data collection form will be piloted to extract data directly from patient paper records in order to estimate the change in clinical practice. Semi-structured interviews and inter-clinic peer workshops will be conducted at 12 months follow-up, and qualitative data collected from these formats will be analysed thematically for explanatory themes that relate to the quantitative findings.

Ethics and Dissemination

Ethical review and approval has been obtained. Findings of the evaluation will be shared in a project report to key stakeholders, presented back to participants, and written into a manuscript for an open access peer-reviewed scientific journal.

STRENGTHS AND LIMITATIONS OF THIS STUDY

- To our knowledge, this is the first description of adapting and piloting WHO essential NCD interventions in primary health care in a low- or middle-income country and provides a methodological example to other jurisdictions
- A mixed methods design allows for a greater understanding of the potential barriers and facilitators to implementation and can inform future health systems development
- Primary health care facilities will be selected from different regions of Republic of Moldova in order to pilot implementation in a variety of contexts throughout the country
- Since this is an evaluation of a pilot implementation, the sample size is based on pragmatism and not statistical power
- We are unable to include patient perspectives and experience in the evaluation, which is an important aspect of health care quality

For peer review only - http://bmjopen.bmj.com/site/about/guidelines.xhtml

INTRODUCTION

Globally, non-communicable diseases (NCDs) account for more than one-half of the global burden of disease.(1) In 2016, an estimated 41 million deaths were due to NCDs, of which nearly half were due to cardiovascular diseases (CVD).(2) Primary health care systems play an important role in the prevention, early detection, and appropriate management of these diseases, but many nations lack primary health care capacity.(3,4)

To support national governments to realize their commitments in reducing the burden of NCDs, as agreed in the United Nations Political Declaration on NCDs, the World Health Assembly endorsed the WHO Global Action Plan for the Prevention and Control of NCDs 2013-2020. To support implementation of this Action Plan, WHO has identified a set of cost-effective policy options ("best buys") for the prevention and control of NCDs within countries.(5)

The Republic of Moldova (henceforth "MDA") is located in Eastern Europe, between Ukraine and Romania; the Capital and largest city is Chisinau. By gross domestic product per capita, MDA is one of the poorest countries in the WHO European Region and it is estimated that 21.9% of citizens live below the absolute poverty line of 1 US Dollar per day.(6)

Non-communicable diseases are a leading cause of death in MDA

While NCDs are a global epidemic, MDA ranks amongst the countries most affected. Nearly 90% of all deaths in MDA are caused by NCDs, the majority of which (55%) are caused by CVD.(7) In 2016, the probability of dying prematurely from any of the four major NCDs (CVDs, cancer, diabetes, chronic respiratory disease) was 24.9%,; almost twice as high for men (33.7%) as women (17.3%).(8) Men and people residing in rural areas are disproportionally impacted by CVD and represent key populations for public health intervention.(7)

This burden is driven by some of the highest rates of NCD risk factors, including tobacco and alcohol use, in the WHO European region indicated by a 2013 STEPS survey.(9) One-in-four (25.3%) Moldovans smoke tobacco and this rate nearly doubles in men.(9) Among adults aged 18 to 69, 61.9% currently consume alcohol and one in five people have engaged in heavy episodic drinking (six or more drinks on any one occasion in the past 30 days).(9)

The overall prevalence of obesity amongst adults is 22.9%, being higher among women (28.5%) as compared with men (17.8%).(7) The prevalence of raised blood pressure (defined as SBP $\geq$ 140 mmHg and/or DBP $\geq$ 90 mmHg or currently taking medication for raised blood pressure) among MDA's adult population is 39.8%, and 76.2% of these patients are not on blood pressure lowering medication.(7) A total of 12.3% of the population have a blood glucose level of $\geq$ 6.1 mmol/L, and 29.4% of the population has a total blood cholesterol level of $\geq$ 5 mmol/L.(7) It is estimated that one in five (23.0%) people aged 40–69 years have a 10-year fatal or non-fatal CVD risk of over 30% (including those with an existing CVD).(7)

Primary health care in MDA and commitment to NCDs

According to the Constitution of Republic of Moldova of 1994, citizens are entitled to a free of charge minimum package of essential health services, including primary health care. However, resource constraints have made it difficult to offer these services and significant gaps in care

For peer review only - http://bmjopen.bmj.com/site/about/guidelines.xhtml

exist.(10) According to the most recent data (2010), there were 5.3 family doctors per 10,000 inhabitants and 25.9 specialist doctors per 10,000 inhabitants. In rural areas these rates are halved, leading to human resource shortages in primary care.(10) Approximately 17% of practicing physicians in MDA work in primary health care, and 92% of them rely on paper clinical records.(6) The most recent estimate (2009) states that there are approximately 630 primary health care centres throughout the country, or 21.2 centres per 100,000 people.(6)

Despite these health system challenges, the Government of Republic of Moldova is committed to improving primary health care capacity for NCDs. It is estimated that 60% of hypertension-related hospital admissions (about 12,000 annually) and 40% of diabetes-related hospitalizations (about 5,000 annually) could be prevented through strengthened primary health care for these conditions.(11)

As such, strengthening primary health care is one of the commitments set out in the Action Program of the Government of Republic of Moldova 2016–2018.(12) To do this requires the development of simplified clinical protocols, in-person training programs for nurses and doctors, and a core set of indicators to monitor and evaluate changes in the quality of care.

Essential interventions to prevent cardiovascular diseases in primary health care

In order to build capacity in primary health care and ultimately prevent premature mortality from CVD in MDA, a study was envisioned to adapt and pilot the World Health Organization Package of Essential NCD Intervention from Primary Healthcare in Low Resource Settings (WHO PEN).(3) WHO PEN includes simplified clinical protocols which together cover the integrated management of hypertension and diabetes, as well as education and counselling on healthy behaviours aimed to prevent CVD. The central strategy of this integrated approach is the use of total cardiovascular risk assessment to stratify and target individuals at high CVD risk, a process considered to be a “best buy” intervention by the WHO.(5)

Since these approaches were unprecedented in MDA, the Ministry of Health, Labour and Social Protection convened a national steering group to lead the adaptation and pilot process, with the goal of using the findings for future health systems development. Led by the primary health care division of the Ministry of Health, the steering group is comprised of representatives from the Nicolae Testemitanu State University of Medicine and Pharmacy and the National Public Health Agency. The national steering group is supported by an international team of experts coordinated jointly by the WHO Regional Office for Europe and WHO Country Office in the Republic of Moldova.

AIM AND OBJECTIVES

Aim

The aim of the evaluation is to determine the feasibility of implementing and evaluating essential interventions for the prevention of cardiovascular disease in primary health care in MDA, with a view toward national scale-up.

Objectives

In order to achieve this aim, the four overarching objectives of the evaluation are to:

For peer review only - http://bmjopen.bmj.com/site/about/guidelines.xhtml

1. Determine the baseline performance of primary health care services with respect to essential interventions for the prevention and management of CVD
2. Assess the ability to implement MDA-adapted WHO PEN protocols one and two in pilot primary health care centres
3. Estimate the change in performance of pilot primary health care centres after 12 months of protocol implementation and compare this to control clinics using usual care
4. Determine the feasibility of collecting quantitative data required for future studies of effectiveness from the existing informal paper clinical record system

METHODS AND ANALYSIS

Overview of Process and Design

An overview of the methods used to adapt, pilot, and evaluate essential interventions for CVD in primary health care in MDA are summarized by the following seven steps.

<u>Step One: Adaptation of WHO PEN Protocols to the National Context</u>

Under the direction of the national steering group, WHO PEN protocols one and two will be compared and contrasted to national disease specific guidelines. The WHO PEN protocols will then be adapted to ensure consistency with the organization, culture, and availability of resources of the health system, while ensuring that they remain simple clinical decision support tools.

<u>Step Two: Development of a Training Package for Primary Health Care Workers</u>

A three-day training package will be developed under the direction of the national steering group in order to provide in-person theoretical and practical training to nurses and doctors working in primary health care. This will include lectures, clinical case studies, and practical exercises that embrace the experience and knowledge of participants.

<u>Step Three: Collection of Baseline Data</u>

According to the Ministry of Health process, a list of 20 primary health care clinics will be nominated and provided to the working group. They will then be randomized into an intervention group arm (n=10) and control arm (n=10). Data for quantitative indicators will be extracted from all 20 clinics by randomly sampling individual paper-based patient records from all primary health care units using a standardized data collection instrument. This will be done by a specially trained group of postgraduate medical trainees.

<u>Step Four: Training Staff in Pilot Clinics</u>

All doctors and nurses from the primary health care centres in the intervention arm will be invited to be trained together by a national team of experts in groups of approximately 30. It is estimated that up to 200 health workers will be trained in total. At the end of training each PHC team will pass through evaluation at the University Centre for Simulation in Medical Training using objective structured clinical exams and get feedback from trainers and peers.

<u>Step Five: Implementation of Protocols</u>

Trained participants from the ten primary health care clinics in the intervention arm will then be free to implement the clinical protocols and change their clinical practice, without incentives, for 12 months. During this time, a team of national experts will be created to offer support (distance

For peer review only

and on-the-job) to the primary health care centres in the intervention arm. All ten clinics in the intervention arm will receive at least one in-person follow-up support visit.

Step Six: Collection of Follow-up Data

After 12 months, using the same method and data collection instruments used to collect baseline quantitative data (Step Three), data will again be extracted from randomly selected individual paper-based patient records from all 20 health care centres. Five primary health care centres from the intervention arm will be selected by the national steering group for one-on-one semi structured interviews with health staff. This will be supplemented by inviting a selection of staff from all ten health centres in the intervention arm to participate in focus groups. Together, these qualitative data will be analysed thematically for explanatory themes.

Step Seven: Evaluation of Results and Sharing Experience

The findings of the quantitative and qualitative analyses will be integrated in a final report and shared with key stakeholders, including health staff from the participating primary health care centres. The results will also be shared at a national conference and in an open-access peer reviewed journal, in order to inform the future development of primary health care capacity in MDA.

Methodological Design

A pragmatic, sequential mixed methods explanatory design, composed of quantitative and qualitative strands of equal weight, will be used (Figure 1). This design was chosen for because it allows for the use of qualitative data to enlighten and explain the quantitative findings, including but not limited to the feasibility of collecting data from paper-based records, the contextual factors affecting guideline implementation, changes in clinical practice, and optimization for the future.

Figure 1. Illustration using the GATE frame structure (13) of the mixed methods evaluation design

A sample size of 20 primary health care centres was chosen because it was seen as a good balance of allowing for variation in clinic geography and demography, while still remaining feasible for the pilot implementation. Half of the centres (n=10) will be randomly allocated to the intervention arm and half (n=10) to the control arm. Baseline data will be collected from both intervention and control clinics, ensuring that baseline data is collected before implementation occurs.

Within clinic comparisons will be used to compare the 12 months before implementation with the 12 months of implementation. Between clinics comparison will be used to compare the intervention clinics with control clinics during the same time period.

Eligibility Criteria for Primary Health Care Centres

Health facilities will be nominated by the Ministry of Health for participation based on the following eligibility criteria: (1) primary health care facilities must be operating in the public sector as legal entities; (2) primary health care facilities must be sampled in a way such that they are geographically distributed evenly across the country; equally from the Central, North and

For peer review only - http://bmjopen.bmj.com/site/about/guidelines.xhtml

Southern regions of MDA; and (3) health facilities must be primary health care centres that are managed by family doctors with no specialist doctors working in the facility.

Randomization

The clinics will be stratified based on the ratio of patients to family doctors to minimize possible confounding by doctor caseload, and then randomized electronically into two groups of 10 primary health care centres.

Comparison

The 10 primary health care centres in the intervention arm will be compared to the 10 primary health care centres in the control arm. The control arm will receive no intervention and proceed with usual care.

Quantitative Indictors

Indicators were developed to balance input and process indicators, such as measurement of risk factors and calculation of risk scores, with output (e.g. prescribing) and outcome (e.g. blood pressure control) indicators. While one of the objectives of this evaluation is to determine the ability to measure these indicators based on routine paper records, we used our existing knowledge of the health system to design indicators which were valuable and likely to be feasible to calculate. Table 1 shows the indicator, the question the indicator seeks to answer, and the respective numerator and denominator definitions which will be used in the calculations.

Table 1. Indicators, their numerators and denominators, and questions the indicators answer

Data Collection and Management

Quantitative Data Collection Tool

A standardized data collection template has been developed for extracting anonymized patient data from individual paper records (Table 2). An online version was also made to allow for data entry on a computer or smartphone. It is estimated to take 15 minutes to extract data from one patient record since the records are made of blank paper with no formal structure or organization of health data.

Table 2. Standardized data collection form used to extract data from individual patient records

Method of Randomly Sampling Patient Records

A random sample of the records of patients aged over 40, who have visited the medical facility within the past 12 months, will be taken. Since medical records in MDA are organized alphabetically on shelves, we created a randomly generated list of alphanumeric combinations that allowed for the selection of patient charts at random. For example, an alphanumeric code of "C24" would correspond to the 24th patient chart in the section of last names starting with the letter C.

The list will be followed in the order that it was generated so as to prevent selection bias. The randomly selected chart will then be checked to see if it meets two inclusion criteria: (1) the patient is aged 18 years or older and (2) the patient visited the health centre within the last 12

For peer review only - http://bmjopen.bmj.com/site/about/guidelines.xhtml

months. If the record meets these criteria, data will then be extracted. If it does not, it will be returned to the shelf and the next alphanumeric code on the randomly generated list will be used. This process will be repeated in each clinic until a sample size of 1.2% of the patient population in each clinic is sampled. This proportion was chosen pragmatically such that the average sample per primary health care centre would equal 100 unique patients.

Data Analysis

The change in indicators from baseline to follow-up will be calculated for intervention clinics and compared with control clinics. Subgroup analysis by age, gender, and other demographic features may be done as deemed appropriate by the national steering committee. All analyses will account for stratified sampling.

Qualitative Data Collection

Follow-up Support Visits

Follow-up visits will be made to each intervention clinic at least once during the implementation timeframe (12 months) to provide ad hoc implementation support. These visits will be conducted by members of the national steering group, who will keep field notes about each visit and provide feedback and support to the health centres. The perspectives gained through follow-up support visits will be used by the national steering group to develop preliminary data collection tools for semi-structured interviews.

Semi-Structured Interviews

A maximum variation sample of half of the intervention clinics (n=5) will be chosen, based on the perceived performance of each clinic by the evaluation steering committee. A pragmatic sample of clinic managers (n=1 per clinic), doctors (n=3 per clinic), and nurses (n=3 per clinic) will be interviewed one-on-one, using a semi-structured format. Interviews will proceed until data saturation has been reached, to a maximum of 30 interviews. After obtaining written, informed consent, interviews will be of 30 to 60 minutes in length, audio recorded, and be transcribed verbatim and analysed thematically using framework thematic analysis.(14)

Focus Group Workshop

Participants from all ten implementation clinics will be invited to a workshop to further collect explanatory qualitative data and to critically reflect on the implementation process. Participants will be a mix of doctors, nurses, and managers from the intervention clinics.

Participants will be placed into small groups based on their profession, and asked to complete a standardized worksheet. Each group will be under the guidance of a facilitator, and emergent themes from one-one-one interviews will be used as prompts to each group. The worksheet will allow for each group to directly comment, modify, or add to the emergent themes, create new themes, and organize themes into categories such as barriers and facilitators.

The resulting qualitative data will be analysed thematically using the framework approach, and used to help explain the findings of the quantitative strand.(14)

For peer review only - http://bmjopen.bmj.com/site/about/guidelines.xhtml

Patient and Public Involvement

Neither patients nor the public were involved in the methodological design.

ETHICS AND DISSEMINATION

Ethical Review and Approval

This project was reviewed by the Research Ethics Committee of the Nicolae Testemitanu State University of Medicine and Pharmacy of the Republic of Moldova and granted permission on 31 May 2017.

Dissemination

Quantitative findings will be summarized and presented back to all intervention clinics during follow-up workshops. A comprehensive project report will be written and shared with key stakeholders. A final report of key findings of the evaluation will be written and submitted to an open access peer-reviewed journal and made available to all study participants so they can use the findings to improve their practice. The findings will be used to evaluate the feasibility of a national scale-up of essential NCD interventions in primary health care in MDA.

REFERENCES

1. Benziger CP, Roth GA, Moran AE. The Global Burden of Disease Study and the Preventable Burden of NCD. Glob Heart. England; 2016 Dec;11(4):393–7.
2. World Health Organization (WHO). World Health Statistics [Internet]. 2018. Available from: http://www.who.int/gho/publications/world_health_statistics/2018/en/
3. World Health Organization (WHO). Package of Essential Noncommunicable (PEN) disease interventions for primary health care in low-resource settings [Internet]. 2013. Available from: http://www.who.int/ncds/management/pen_tools/en/
4. World Health Organization (WHO). Global action plan for the prevention and control of NCDs 2013-2020. 2013.
5. Organization WH. Tackling NCDs: "Best buys" and other recommended interventions for the prevention and control of noncommunicable diseases [Internet]. 2017. Available from: http://apps.who.int/iris/bitstream/handle/10665/259232/WHO-NMH-NVI-17.9-eng.pdf;jsessionid=074ED8768EA1FFD0E632DB861E91A6FD?sequence=1
6. World Health Organization (WHO). Evaluation of the sturcutre and provision of pirmary care in the Republic of Moldova. 2012.
7. National Center for Health Management. Health Yearbook: Public Health in Moldova [Internet]. 2015. Available from: http://www.cnms.md/ro/rapoarteo
8. World Health Organization. Global Health Observatory data repository [Internet]. [cited 2018 Jul 10]. Available from: http://apps.who.int/gho/data/node.home
9. World Health Organization (WHO). Prevalence of Noncommunicable Disease Risk Factors in the Republic of Moldova (STEPS) [Internet]. 2014. Available from: http://www.who.int/ncds/surveillance/steps/Moldova_2013_STEPS_Report.pdf
10. Turcanu C, Domente S, Buga M, Richardson E. Health Systems in Transition: Republic of Moldova [Internet]. 2012. Available from: http://www.euro.who.int/__data/assets/pdf_file/0006/178053/HiT-Moldova.pdf
11. World Health Organization (WHO). Ambulatory care sensitive conditions in the Republic of Moldova [Internet]. 2015. Available from:

For peer review only - http://bmjopen.bmj.com/site/about/guidelines.xhtml

http://www.euro.who.int/en/countries/republic-of-moldova/publications/ambulatory-care-sensitive-conditions-in-the-republic-of-moldova-2015

12. Government of the Republic of Moldova. Action Program of the Government of the Republic of Moldova 2016–2018 [Internet]. 2016. Available from: http://gov.md/sites/default/files/document/attachments/guvernul_republicii_moldova_-_programul_de_activitate_al_guvernului_republicii_moldova_2016-2018.pdf

13. Jackson R, Ameratunga S, Broad J, Connor J, Lethaby A, Robb G, et al. The GATE frame: critical appraisal with pictures. Vol. 144, ACP journal club. United States; 2006. p. A8-11.

14. Gale NK, Heath G, Cameron E, Rashid S, Redwood S. Using the framework method for the analysis of qualitative data in multi-disciplinary health research. BMC Med Res Methodol. England; 2013 Sep;13:117.

AUTHORS AND CONTRIBUTIONS

DC, AC, TL, GC, VS, TZ, AA, and JF contributed to the methodological design. DC, AC, TL, and JF contributed to writing the manuscript.

FUNDING STATEMENT

This study is funded jointly by the Swiss Agency for Development and Cooperation (SDC) and WHO Regional Office for Europe.

COMPETING INTERESTS STATEMENT

DC, AC, TL, GC, VS, TZ, AA, JF declare no competing interests.

For peer review only - http://bmjopen.bmj.com/site/about/guidelines.xhtml

Table 1. Indicators, their numerators and denominators, and questions the indicators answer

Question	Indicator	Numerator	Denominator
Are risk factors being measured?	Proportion of eligible patients who have all risk factor values recorded as required for calculation of risk score	Patients aged 40 or older who have visited in the last 12 months who have all measurements required for calculation of risk score within 12 months of the most recent date of visit	Patients aged 40 or older who have visited in the last 12 months
Are risk factor measurements being converted to a total risk score?	Proportion of patients aged 40 or older who have visited in the last 12 months who have all measurements required for calculation of risk score within 12 months of the most recent date of visit, that have a documented risk score	Patients aged 40 or older who have visited in the last 12 months who have all measurements required for calculation of risk score within 12 months of the most recent date of visit, that have a documented risk score	Patients aged 40 or older who have visited in the last 12 months who have all measurements required for calculation of risk score within 12 months of the most recent date of visit
Are risk scores calculated correctly?	Proportion of patients aged 40 or older who have visited in the last 12 months who have all measurements required for calculation of risk score within 12 months of the most recent date of visit, that have a documented risk score that is correct	Patients aged 40 or older who have visited in the last 12 months who have all measurements required for calculation of risk score within 12 months of the most recent date of visit, that have a documented risk score that is correct	Patients aged 40 or older who have visited in the last 12 months who have all measurements required for calculation of risk score within 12 months of the most recent date of visit, that have a documented risk score
Are patients being risk scored?	Proportion of eligible patients with a documented risk score	Patients aged 40 or older who have visited in the last 12 months with a documented risk score	Patients aged 40 or older who have visited in the last 12 months
Are risk scores calculated correctly?	Proportion of eligible patients with a documented risk score that is correct	Patients aged 40 or older who have visited in the last 12 months with a documented risk score that is correct	Patients aged 40 or older who have visited in the last 12 months with a documented risk score
Are statins prescribed to the correct patients?	Proportion of eligible patients prescribed a statin	Patients with existing CVD, diabetics 40 or older with high LDL values (as defined based on total CVD risk of SCORE 10-14% in LDL ≥2.6 mmol/L; with very high risk SCORE ≥15% in LDL ≥1.8 mmol/L), or patients with a SCORE of ≤ 9% and LDL ≥ 2.6 or total cholesterol ≥7.2, or patients with a SCORE of 10-14% and a LDL >=1.8 or total cholesterol ≥7.2 mmol/L, or patients with a SCORE of ≥15%, prescribed a statin	Patients with existing CVD, diabetics 40 or older with high LDL values (as defined based on total CVD risk of SCORE 10-14% in LDL ≥2.6 mmol/L; with very high risk SCORE ≥15% in LDL ≥1.8 mmol/L), or patients with a SCORE of ≤ 9% and LDL ≥2.6 or total cholesterol ≥ 7.2, or patients with a SCORE of 10-14% and a LDL ≥1.8 or total cholesterol ≥7.2 mmol/L, or patients with a SCORE of ≥15%
Are statins prescribed correctly based on documented risk score?	Proportion of patients eligible based on documented risk score prescribed a statin	Patients with a documented risk score as very high risk SCORE ≥15% prescribed a statin	Patients with a documented risk score as very high risk SCORE ≥15%
Are patients with existing disease, who do not require the calculation of a risk score to prescribe satins, prescribed statins?	Proportion of patients with existing CVD prescribed a statin	Patients with existing CVD prescribed a statin	Patients with existing CVD
Is the blood pressure of high risk patients controlled?	Proportion of high risk patients (SCORE ≥15% or DM and age over 40)	Patients with a true risk score indicating a very high risk (SCORE ≥15%) or DM and age	Patients with a true risk score indicating a very high risk (SCORE ≥15%) or DM and

For peer review only - http://bmjopen.bmj.com/site/about/guidelines.xhtml

	whose last two recorded blood pressure measurements were <130/80 mmHg	over 40 whose last two documented blood pressure readings were <130/80	age over 40
Is the blood pressure of lower risk patients controlled?	Proportion of lower risk patients (SCORE<15%) whose last two recorded blood pressure measurements were <140/90 mmHg	Patients with a true risk score indicating <15% whose last two documented blood pressure readings were <140/90	Patients with a true risk score indicating <15%
Are patients with existing CVD prescribed basic medications to reduce risk?	Proportion of patients with existing CVD prescribed a statin and aspirin and blood pressure lowering treatment	Patients with existing CVD prescribed a statin and aspirin and blood pressure lowering treatment	Patients with existing CVD
Is the blood glucose of diabetic patients controlled?	Proportion of diabetic patients with glycaemic control as defined by last two HbA1c measurements	Patients with diabetes 2 whose last two HbA1c measurements were below personal target as defined by MDA adapted WHO PEN 1	Patients with diabetes type 2
Is the blood pressure of hypertensive patients controlled?	Proportion of confirmed hypertensive patients whose SBP is <140/90 at last two visits	Patients with confirmed hypertension whose last two blood pressure readings were <140/90	Patients with confirmed hypertension
What is the prevalence of high blood pressure?	Proportion of people whose last two systolic blood pressure reading are 140 mmHg or above	Patients whose last two systolic blood pressure readings were ≥140	All patients over 18

For peer review only

413 **Table 2.** Standardized data collection form used to extract data from individual patient records
414

Data Collection Question	Answer
What is your name? (Name of person extracting data)	
Date of Data Extraction (MM-DD-YYYY)	
Write the Clinic Name	
Is this a duplicate extraction?	
If it is a duplicate extraction, enter the number you and your extraction partner have assigned to this file.	
Date of Birth (MM-DD-YYYY)	
Sex (M/F)	
Smoking Status (Y/M)	
Diagnosis of Hypertension (Y/N)	
Date of Hypertension Diagnosis (MM-DD-YYYY)	
Can you find one or more blood pressure readings? (Y/N)	
Most Recent Systolic Blood Pressure	
Most Recent Diastolic Blood Pressure	
Date of the Most Recent Blood Pressure Measurement (MM-DD-YYYY)	
Can you find a second most recent blood pressure reading? (Y/N)	
Second most recent systolic blood pressure	
Second most recent diastolic blood pressure	
Date of the second most recent systolic blood pressure (MM-DD-YYYY)	
Diagnosis of Diabetes (Type 1, Type 2, No)	
Can you find one or more HbA1c measurements? (Y/N)	
Most recent HbA1c reading (mmol/mol)	
Date of the most recent HbA1c measurement? (MM-DD-YYYY)	
Can you find another HbA1c measurement? (Y/N)	
Second most recent HbA1c reading (mmol/mol, otherwise specify unit)	
Date of the second most recent HbA1c reading? (MM-DD-YYYY)	
Can you find one or more total cholesterol measurements? (Y/N)	
Most recent total cholesterol reading (mmol/L)	
Date of the most recent cholesterol reading (MM-DD-YYYY)	
Can you find another cholesterol measurement? (Y/N)	
Second most recent cholesterol reading (mmol/L)	
Date of the second most recent cholesterol reading (MM-DD-YYYY)	
Was the patient prescribed a statin? (Y/N)	
What was the date of the statin prescription? (MM-DD-YYYY)	

For peer review only

What was the drug and dose?	
Does the patient have existing CVD? (Y/N)	
State the type of CVD	
Has the patient been prescribed acetylsalicylic acid (ASA or aspirin)? (Y/N)	
What was the most recent date that ASA was prescribed? (MM-DD-YYYY)	
Has the patient been prescribed anti-hypertensives? (Y/N)	
What was the most recent date that anti-hypertensives were prescribed? (MM-DD-YYYY)	
Can you find a documented ESC SCORE risk score? (Y/N)	
Enter the most recent documented ESC SCORE risk score (%)	
What was the date the risk score was documented? (MM-DD-YYYY)	
Please record any important notes about the data extraction here. Examples include an error you think may have been made, clarification of the units for measurements (e.g. mmol/L vs mg/dL). Or notes that you would like for yourself.	

For peer review only

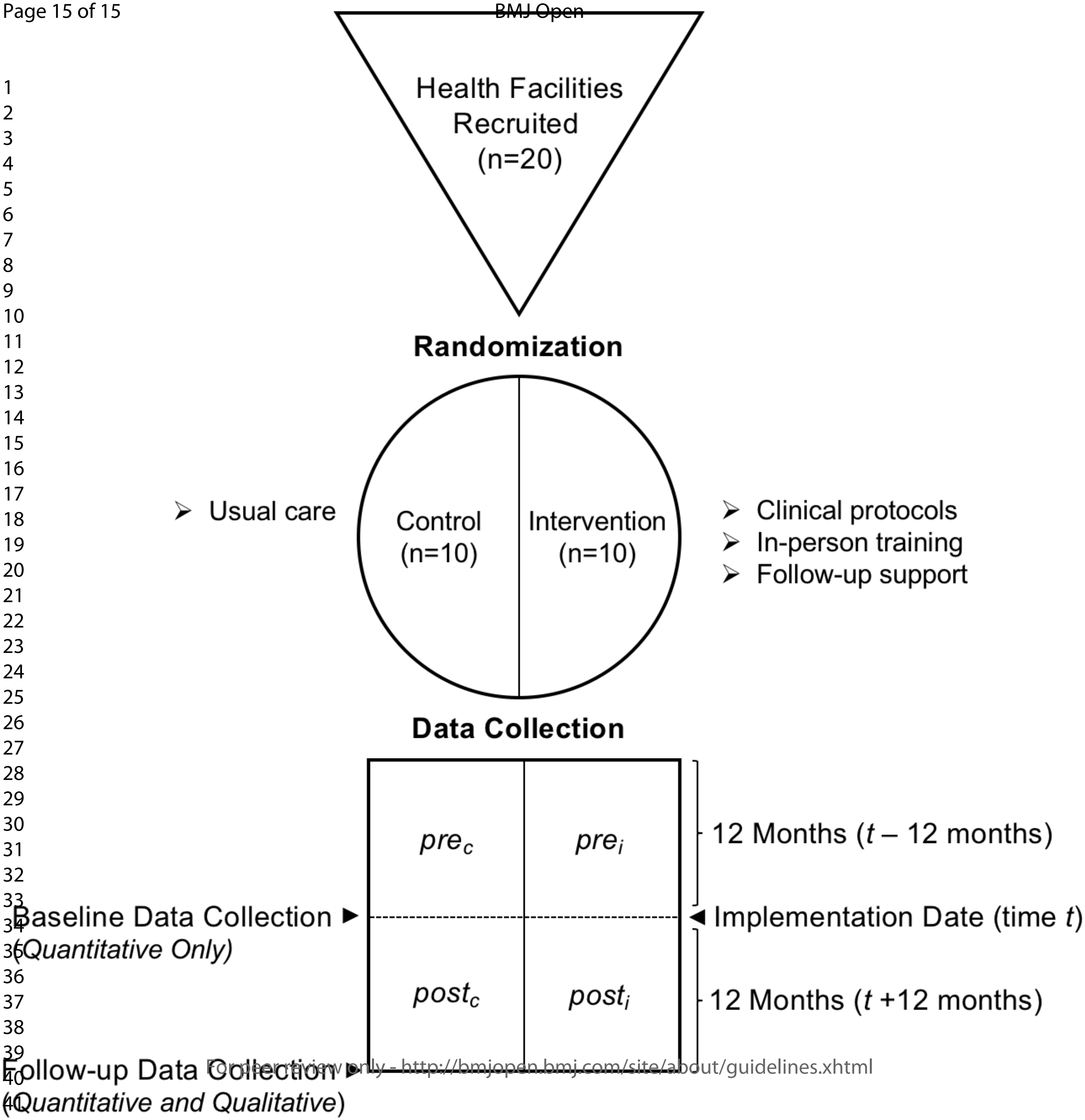
Health Facilities Recruited (n=20)
Randomization
Usual care
Control (n=10)
Intervention (n=10)
Clinical protocols
In-person training
Follow-up support
Data Collection
pre_c
pre_i
12 Months (t – 12 months)
Baseline Data Collection
(Quantitative Only)
Implementation Date (time t)
post_c
post_i
12 Months (t +12 months)
Follow-up Data Collection
(Quantitative and Qualitative)

BMJ Open

Protocol for the Evaluation of a Pilot Implementation of Essential Interventions for the Prevention of Cardiovascular Diseases in Primary Health Care in the Republic of Moldova

Journal:	*BMJ Open*
Manuscript ID	bmjopen-2018-025705.R1
Article Type:	Protocol
Date Submitted by the Author:	23-Mar-2019
Complete List of Authors:	Collins, Dylan; University of British Columbia,<br>Ciobanu, Angela<br>Laatikainen, Tiina; National Institute for Health and Welfare, Epidemiology and Health Promotion<br>Curocichin, Ghenadie<br>Salaru, Virginia<br>Zatic, Tatiana<br>Anisei, Angela<br>Farrington, Jill
**Primary Subject Heading**:	Cardiovascular medicine
Secondary Subject Heading:	Public health
Keywords:	Cardiology < INTERNAL MEDICINE, PRIMARY CARE, Quality in health care < HEALTH SERVICES ADMINISTRATION & MANAGEMENT, Protocols & guidelines < HEALTH SERVICES ADMINISTRATION & MANAGEMENT

SCHOLARONE™
Manuscripts

Protocol for the Evaluation of a Pilot Implementation of Essential Interventions for the Prevention of Cardiovascular Diseases in Primary Health Care in the Republic of Moldova

Dylan R. J. Collins[1], Angela Ciobanu[2], Tiina Laatikainen[3], Ghenadie Curocichin[4], Virginia Salaru[4], Tatiana Zatic[5], Angela Anisei[6], Jill L. Farrington[7]

[1]University of British Columbia, Vancouver, Canada
[2]World Health Organization Country Office in Republic of Moldova, Chisinau, Republic of Moldova
[3]Institute of Public Health and Clinical Nutrition, Helsinki, Finland
[4]Family Medicine Department, Nicolae Testemitanu State Medical and Pharmaceutical University, Chisinau, Republic of Moldova
[5]Primary, Emergency and Community Health Policies Department, Ministry of Health, Labour and Social Protection, Chisinau, Republic of Moldova
[6]Department on Quality Management of Health Services, National Public Health Agency, Republic of Moldova
[7]World Health Organization Regional Office for Europe, Copenhagen, Denmark

Correspondence to Dr. Dylan Collins at dylan.collins@alumni.ubc.ca

For peer review only

ABSTRACT

Introduction

Nearly 90% of all deaths in Republic of Moldova are caused by NCDs, the majority of which (55%) are caused by CVD. In addition to reducing premature mortality from CVD, it is estimated that strengthening primary health care could cut the number hypertension-related hospital admissions and diabetes-related hospitalizations in half. The aim of this evaluation is to determine the feasibility of implementing and evaluating essential interventions for the prevention of CVD in primary health care in Republic of Moldova, with a view toward national scale-up.

Methods and Analysis

A national steering group including international experts will be convened to adapt WHO PEN protocols one and two to the health system of Republic of Moldova, develop and conduct training of primary health care workers, and test a core set of indicators to monitor the quality of care and change in clinical practice. To evaluate the impact of this pilot implementation, a pragmatic, sequential mixed methods explanatory design, composed of quantitative and qualitative strands of equal weight, will be used. Twenty primary health care centres will be selected and randomized to the training and implementation arm (n=10) and the usual care arm (n=10). At baseline and 12 months follow-up, a standardized data collection form will be piloted to extract data directly from patient paper records in order to estimate the change in clinical practice. Semi-structured interviews and inter-clinic peer workshops will be conducted at 12 months follow-up, and qualitative data collected from these formats will be analysed thematically for explanatory themes that relate to the quantitative findings.

Ethics and Dissemination

Ethical review and approval has been obtained. Findings of the evaluation will be shared in a project report to key stakeholders, presented back to participants, and written into a manuscript for an open access peer-reviewed scientific journal.

STRENGTHS AND LIMITATIONS OF THIS STUDY

- To our knowledge, this is the first description of adapting and piloting WHO essential NCD interventions in primary health care in a low- or middle-income country and provides a methodological example to other jurisdictions
- A mixed methods design allows for a greater understanding of the potential barriers and facilitators to implementation and can inform future health systems development
- Primary health care facilities will be selected from different regions of Republic of Moldova in order to pilot implementation in a variety of contexts throughout the country
- Since this is an evaluation of a pilot implementation, the sample size is based on pragmatism and not statistical power
- We are unable to include patient perspectives and experience in the evaluation, which is an important aspect of health care quality

For peer review only - http://bmjopen.bmj.com/site/about/guidelines.xhtml

INTRODUCTION

Globally, non-communicable diseases (NCDs) account for more than one-half of the global burden of disease.(1) In 2016, an estimated 41 million deaths were due to NCDs, of which nearly half were due to cardiovascular diseases (CVD).(2) Primary health care systems play an important role in the prevention, early detection, and appropriate management of these diseases, but many nations lack primary health care capacity.(3,4)

To support national governments to realize their commitments in reducing the burden of NCDs, as agreed in the United Nations Political Declaration on NCDs, the World Health Assembly endorsed the WHO Global Action Plan for the Prevention and Control of NCDs 2013-2020. To support implementation of this Action Plan, WHO has identified a set of cost-effective policy options ("best buys") for the prevention and control of NCDs within countries.(5)

The Republic of Moldova (henceforth "MDA") is located in Eastern Europe, between Ukraine and Romania; the Capital and largest city is Chisinau. By gross domestic product per capita, MDA is one of the poorest countries in the WHO European Region and it is estimated that 21.9% of citizens live below the absolute poverty line of 1 US Dollar per day.(6)

Non-communicable diseases are a leading cause of death in MDA

While NCDs are a global epidemic, MDA ranks amongst the countries most affected. Nearly 90% of all deaths in MDA are caused by NCDs, the majority of which (55%) are caused by CVD.(7) In 2016, the probability of dying prematurely from any of the four major NCDs (CVDs, cancer, diabetes, chronic respiratory disease) was 24.9%,; almost twice as high for men (33.7%) as women (17.3%).(8) Men and people residing in rural areas are disproportionally impacted by CVD and represent key populations for public health intervention.(7)

This burden is driven by some of the highest rates of NCD risk factors, including tobacco and alcohol use, in the WHO European region indicated by a 2013 STEPS survey.(9) One-in-four (25.3%) Moldovans smoke tobacco and this rate nearly doubles in men.(9) Among adults aged 18 to 69, 61.9% currently consume alcohol and one in five people have engaged in heavy episodic drinking (six or more drinks on any one occasion in the past 30 days).(9)

The overall prevalence of obesity amongst adults is 22.9%, being higher among women (28.5%) as compared with men (17.8%).(9) The prevalence of raised blood pressure (defined as SBP $\geq$ 140 mmHg and/or DBP $\geq$ 90 mmHg or currently taking medication for raised blood pressure) among MDA's adult population is 39.8%, and 76.2% of these patients are not on blood pressure lowering medication.(9) A total of 12.3% of the population have a blood glucose level of $\geq$ 6.1 mmol/L, and 29.4% of the population has a total blood cholesterol level of $\geq$ 5 mmol/L.(9) It is estimated that one in five (23.0%) people aged 40–69 years have a 10-year fatal or non-fatal CVD risk of over 30% (including those with an existing CVD).(9)

Primary health care in MDA and commitment to NCDs

According to the Constitution of Republic of Moldova of 1994, citizens are entitled to a free of charge minimum package of essential health services, including primary health care. However, resource constraints have made it difficult to offer these services and significant gaps in care

For peer review only

exist.(10) According to the most recent data (2010), there were 5.3 family doctors per 10,000 inhabitants and 25.9 specialist doctors per 10,000 inhabitants. In rural areas these rates are halved, leading to human resource shortages in primary care.(10) Approximately 17% of practicing physicians in MDA work in primary health care, and 92% of them rely on paper clinical records.(6) The most recent estimate (2009) states that there are approximately 630 primary health care centres throughout the country, or 21.2 centres per 100,000 people.(6)

Despite these health system challenges, the Government of Republic of Moldova is committed to improving primary health care capacity for NCDs. It is estimated that 60% of hypertension-related hospital admissions (about 12,000 annually) and 40% of diabetes-related hospitalizations (about 5,000 annually) could be prevented through strengthened primary health care for these conditions, including better identification and management of those at increased CVD risk.(11)

Given the need and international policy support for addressing this gap in NCD care, there was a favourable window of opportunity to act with impact. As such, strengthening primary health care was set out as one of the main commitments in the Action Program of the Government of Republic of Moldova 2016–2018.(12) To do this requires the development of simplified clinical protocols, in-person training programs for nurses and doctors, and a core set of indicators to monitor and evaluate changes in the quality of care.

Essential interventions to prevent cardiovascular diseases in primary health care

In order to build capacity in primary health care and ultimately prevent premature mortality from CVD in MDA, a study was envisioned to adapt and pilot the World Health Organization Package of Essential NCD Intervention from Primary Healthcare in Low Resource Settings (WHO PEN).(3) WHO PEN includes simplified clinical protocols which together cover the integrated management of hypertension and diabetes, as well as education and counselling on healthy behaviours aimed to prevent CVD. The central strategy of this integrated approach is the use of total cardiovascular risk assessment to stratify and target individuals at high CVD risk, a process considered to be a “best buy” intervention by the WHO.(5)

These interventions are aimed at tackling areas identified in a 2014 WHO assessment of challenges and opportunities for better NCD outcomes in Moldova. (13) This includes shortcomings amongst health workers in the identification and management of individuals with increased cardiovascular risk. The interventions are expected to add to the current quality of care by targeting interventions (non-pharmacological and/or pharmacological) to those at highest risk who stand to gain the most in absolute cardiovascular risk reduction, while also emphasizing improvements in the organization of care. The intervention also includes practical face-to-face training and follow-up implementation support. Current practice underutilizes these medical strategies and guidelines (e.g. CVD risk score directed primary prevention), in addition to limited task sharing with non-physician health works (e.g. nurses) in these care pathways. (13) At the study’s inception, there were no known developments beyond the scope of this project that could change clinical practice for NCDs in primary health care.

Since the use of WHO PEN was unprecedented in MDA, the Ministry of Health, Labour and Social Protection convened a national steering group to lead the adaptation and pilot process, with the goal of using the findings for future health systems development. Led by the primary health care

For peer review only

division of the Ministry of Health, the steering group is comprised of representatives from the Nicolae Testemitanu State University of Medicine and Pharmacy and the National Public Health Agency. The national steering group is supported by an international team of experts coordinated jointly by the WHO Regional Office for Europe and WHO Country Office in the Republic of Moldova.

AIM AND OBJECTIVES

Aim

The aim of the evaluation is to determine the feasibility of implementing and evaluating essential interventions for the prevention of cardiovascular disease in primary health care in MDA, with a view toward national scale-up.

Objectives

Primary Objectives

1. Assess the ability to implement MDA-adapted WHO PEN protocols one and two in pilot primary health care centres
2. Determine the feasibility of collecting quantitative data required for future studies of effectiveness from the existing informal paper clinical record system

Secondary Objectives

1. Determine the baseline performance of primary health care services with respect to essential interventions for the prevention and management of CVD
2. Estimate the change in performance of pilot primary health care centres after 12 months of protocol implementation and compare this to control clinics using usual care

METHODS AND ANALYSIS

Overview of Process and Design

An overview of the methods used to adapt, pilot, and evaluate essential interventions for CVD in primary health care in MDA are summarized by the following seven steps, which are planned to occur from September 2016 to May 2019.

Step One: Adaptation of WHO PEN Protocols to the National Context

Under the direction of the national steering group, WHO PEN protocols one and two will be compared and contrasted to national disease specific guidelines. The WHO PEN protocols will then be adapted to ensure consistency with the organization, culture, and availability of resources of the health system, while ensuring that they remain simple clinical decision support tools.

Step Two: Development of a Training Package for Primary Health Care Workers

A three-day training package will be developed under the direction of the national steering group in order to provide in-person theoretical and practical training to nurses and doctors working in primary health care. This will include lectures, clinical case studies, and practical exercises that embrace the experience and knowledge of participants.

Step Three: Collection of Baseline Data

For peer review only

According to the Ministry of Health process, a list of 20 primary health care clinics will be nominated and provided to the working group. They will then be randomized into an intervention group arm (n=10) and control arm (n=10). Data for quantitative indicators will be extracted from all 20 clinics by randomly sampling individual paper-based patient records from all primary health care units using a standardized data collection instrument. This will be done before randomization by a specially trained group of postgraduate medical trainees, such that neither the clinics nor the data extractors will know the allocation of each clinic to intervention or control arm.

<u>Step Four: Training Staff in Pilot Clinics</u>

All doctors and nurses from the primary health care centres in the intervention arm will be invited to be trained together by a national team of experts in groups of approximately 30. It is estimated that up to 200 health workers will be trained in total. At the end of training each PHC team will pass through evaluation at the University Centre for Simulation in Medical Training using objective structured clinical exams and get feedback from trainers and peers.

<u>Step Five: Implementation of Protocols</u>

Trained participants from the ten primary health care clinics in the intervention arm will then be free to implement the clinical protocols and change their clinical practice, without incentives, for 12 months. During this time, a team of national experts will be created to offer support (distance and on-the-job) to the primary health care centres in the intervention arm. All ten clinics in the intervention arm will receive at least one in-person follow-up support visit.

<u>Step Six: Collection of Follow-up Data</u>

After 12 months, using the same method and data collection instruments used to collect baseline quantitative data (Step Three), data will again be extracted from randomly selected individual paper-based patient records from all 20 health care centres. Five primary health care centres from the intervention arm will be selected by the national steering group for one-on-one semi structured interviews with health staff. This will be supplemented by inviting a selection of staff from all ten health centres in the intervention arm to participate in focus groups. Together, these qualitative data will be analysed thematically for explanatory themes.

<u>Step Seven: Evaluation of Results and Sharing Experience</u>

The findings of the quantitative and qualitative analyses will be integrated in a final report and shared with key stakeholders, including health staff from the participating primary health care centres. The results will also be shared at a national conference and in an open-access peer reviewed journal, in order to inform the future development of primary health care capacity in MDA.

Methodological Design

A pragmatic, sequential mixed methods explanatory design, composed of quantitative and qualitative strands of equal weight, will be used (Figure 1). This design was chosen because it allows for the use of qualitative data to enlighten and explain the quantitative findings, including but not limited to the feasibility of collecting data from paper-based records, the contextual factors affecting guideline implementation, changes in clinical practice, and optimization for the future.

Figure 1. Illustration using the GATE frame structure (14) of the mixed methods evaluation design

For peer review only

A sample size of 20 primary health care centres was chosen because it was seen as a good balance of allowing for variation in clinic geography and demography, while still remaining feasible for the pilot implementation. Half of the centres (n=10) will be randomly allocated to the intervention arm and half (n=10) to the control arm. Baseline data will be collected from both intervention and control clinics, ensuring that baseline data is collected before implementation occurs.

Within clinic comparisons will be used to compare the 12 months before randomization with the 12 months of implementation. Between clinics comparison will be used to compare the intervention clinics with control clinics during the same time period.

Eligibility Criteria for Primary Health Care Centres
Health facilities will be nominated by the Ministry of Health for participation based on the following eligibility criteria: (1) primary health care facilities must be operating in the public sector as legal entities; (2) primary health care facilities must be sampled in a way such that they are geographically distributed evenly across the country; equally from the Central, North and Southern regions of MDA; and (3) health facilities must be primary health care centres that are managed by family doctors with no specialist doctors working in the facility. These criteria were chosen in order to select a group of clinics that sufficiently reflect the majority of primary health care facilities in Moldova.

Randomization
The clinics will be stratified based on the ratio of patients to family doctors to minimize possible confounding by doctor caseload, and then randomized electronically into two groups of 10 primary health care centres.

Comparison
The 10 primary health care centres in the intervention arm will be compared to the 10 primary health care centres in the control arm. The control arm will receive no intervention and proceed with usual care.

Quantitative Indictors
Indicators were developed to balance input and process indicators, such as measurement of risk factors and calculation of risk scores, with output (e.g. prescribing) and outcome (e.g. blood pressure control) indicators. While one of the objectives of this evaluation is to determine the ability to measure these indicators based on routine paper records, we used our existing knowledge of the health system to design indicators which were valuable and likely to be feasible to calculate. Table 1 shows the indicator, the question the indicator seeks to answer, and the respective numerator and denominator definitions which will be used in the calculations.

Table 1. Indicators, their numerators and denominators, and questions the indicators answer

Data Collection and Management

<u>Quantitative Data Collection Tool</u>

For peer review only

For peer review only - http://bmjopen.bmj.com/site/about/guidelines.xhtml

A standardized data collection template has been developed for extracting anonymized patient data from individual paper records (Table 2). An online version was also made to allow for data entry on a computer or smartphone. It is estimated to take 15 minutes to extract data from one patient record since the records are made of blank paper with no formal structure or organization of health data.

Table 2. Standardized data collection form used to extract data from individual patient records

Method of Randomly Sampling Patient Records

A random sample of the records of patients aged over 18, who have visited the medical facility within the past 12 months, will be taken. Since medical records in MDA are organized alphabetically on shelves, we created a randomly generated list of alphanumeric combinations that allowed for the selection of patient charts at random. For example, an alphanumeric code of "C24" would correspond to the 24th patient chart in the section of last names starting with the letter C.

The list will be followed in the order that it was generated so as to prevent selection bias. The randomly selected chart will then be checked to see if it meets two inclusion criteria: (1) the patient is aged 18 years or older and (2) the patient visited the health centre within the last 12 months. If the record meets these criteria, data will then be extracted. If it does not, it will be returned to the shelf and the next alphanumeric code on the randomly generated list will be used. This process will be repeated in each clinic until a sample size of 1.2% of the patient population in each clinic is sampled. This proportion was chosen pragmatically such that the average sample per primary health care centre would equal 100 unique patients.

Data Analysis

The change in indicators from baseline to follow-up will be calculated for intervention clinics and compared with control clinics (Table 1). Subgroup analysis by age, gender, and other demographic features may be done as deemed appropriate by the national steering committee. All analyses will account for stratified sampling. Since the health centre is the unit of inference for the outcomes (e.g. health centre proportion of eligible patients with a documented CVD risk score), use of an intracluster correlation coefficient is not required for analyses of these outcomes. Age and gender adjusted logistic regression models will be used to analyse the differences in pre-defined indicators between intervention and control clinics and between baseline and follow-up. The differences in means of continuous variables between the intervention and control clinics and baseline and follow-up will be analysed using age and gender adjusted analysis of variance.

Qualitative Data Collection

Follow-up Support Visits

Follow-up visits will be made to each intervention clinic at least once during the implementation timeframe (12 months) to provide ad hoc implementation support. These visits will be conducted by members of the national steering group, who will keep field notes about each visit and provide feedback and support to the health centres. The perspectives gained through follow-up support visits will be used by the national steering group to develop preliminary data collection tools for semi-structured interviews.

For peer review only - http://bmjopen.bmj.com/site/about/guidelines.xhtml

Semi-Structured Interviews

A maximum variation sample of half of the intervention clinics (n=5) will be chosen, based on the perceived performance of each clinic by the evaluation steering committee. A pragmatic sample of clinic managers (n=1 per clinic), doctors (n=3 per clinic), and nurses (n=3 per clinic) will be interviewed one-on-one, using a semi-structured format. Interviews will proceed until data saturation has been reached, to a maximum of 30 interviews. After obtaining written, informed consent, interviews will be of 30 to 60 minutes in length, audio recorded, and be transcribed verbatim and analysed thematically using framework thematic analysis.(15) The interviews will be conducted by members of the steering group, but the interviewers will be allocated to participants from health centres with whom they did not provide follow-up support visits.

Focus Group Workshop

Participants from all ten implementation clinics will be invited to a workshop to further collect explanatory qualitative data and to critically reflect on the implementation process. Participants will be a mix of doctors, nurses, and managers from the intervention clinics.

Participants will be placed into small groups based on their profession, and asked to complete a standardized worksheet. Each group will be under the guidance of a facilitator, and emergent themes from one-one-one interviews will be used as prompts to each group. The worksheet will allow for each group to directly comment, modify, or add to the emergent themes, create new themes, and organize themes into categories such as barriers and facilitators.

Integration of Quantitative and Qualitative Strands

The resulting qualitative data will be analysed thematically using the framework approach, and used to help explain the findings of the quantitative strand.(15) Following the sequential mixed method design, integration of the qualitative findings with quantitative findings will allow for the interpretation of the results in light of each other. This may include post-hoc analysis of effectiveness of some of the quantitative outcomes as appropriate, to further add meaning to the integration of qualitative and quantitative strands.

Patient and Public Involvement

Neither patients nor the public were involved in the methodological design.

ETHICS AND DISSEMINATION

Ethical Review and Approval

This project was reviewed by the Research Ethics Committee of the Nicolae Testemitanu State University of Medicine and Pharmacy of the Republic of Moldova and granted permission on 31 May 2017.

Dissemination

Quantitative findings will be summarized and presented back to all intervention clinics during follow-up workshops. A comprehensive project report will be written and shared with key stakeholders. A final report of key findings of the evaluation will be written and submitted to an open access peer-reviewed journal and made available to all study participants so they can use the findings to improve their practice. The findings will be used to evaluate the feasibility of a national scale-up of essential NCD interventions in primary health care in MDA.

For peer review only - http://bmjopen.bmj.com/site/about/guidelines.xhtml

REFERENCES

1. Benziger CP, Roth GA, Moran AE. The Global Burden of Disease Study and the Preventable Burden of NCD. Glob Heart. 2016 Dec;11(4):393–7.
2. World Health Organization (WHO). World Health Statistics [Internet]. 2018. Available from: http://www.who.int/gho/publications/world_health_statistics/2018/en/
3. World Health Organization (WHO). Package of Essential Noncommunicable (PEN) disease interventions for primary health care in low-resource settings [Internet]. 2013. Available from: http://www.who.int/ncds/management/pen_tools/en/
4. World Health Organization (WHO). Global action plan for the prevention and control of NCDs 2013-2020. 2013.
5. Organization WH. Tackling NCDs: “Best buys” and other recommended interventions for the prevention and control of noncommunicable diseases [Internet]. 2017. Available from: http://www.who.int/ncds/management/best-buys/en/
6. World Health Organization (WHO). Evaluation of the sturcutre and provision of pirmary care in the Republic of Moldova. 2012.
7. National Center for Health Management. Health Yearbook: Public Health in Moldova [Internet]. 2015. Available from: http://www.cnms.md/ro/rapoarteo
8. World Health Organization. Global Health Observatory data repository [Internet]. [cited 2018 Jul 10]. Available from: http://apps.who.int/gho/data/node.home
9. World Health Organization (WHO). Prevalence of Noncommunicable Disease Risk Factors in the Republic of Moldova (STEPS) [Internet]. 2014. Available from: http://www.who.int/ncds/surveillance/steps/Moldova_2013_STEPS_Report.pdf
10. Turcanu C, Domente S, Buga M, Richardson E. Health Systems in Transition: Republic of Moldova [Internet]. 2012. Available from: http://www.euro.who.int/__data/assets/pdf_file/0006/178053/HiT-Moldova.pdf
11. World Health Organization (WHO). Ambulatory care sensitive conditions in the Republic of Moldova [Internet]. 2015. Available from: http://www.euro.who.int/en/countries/republic-of-moldova/publications/ambulatory-care-sensitive-conditions-in-the-republic-of-moldova-2015
12. Government of the Republic of Moldova. Action Program of the Government of the Republic of Moldova 2016–2018 [Internet]. 2016. Available from: http://gov.md/sites/default/files/document/attachments/guvernul_republicii_moldova_-_programul_de_activitate_al_guvernului_republicii_moldova_2016-2018.pdf
13. World Health Organization Regional Office for Europe. Better noncommunicable disease outcomes: challenges and opportunities for health systems -- Country Assessment of Republic of Moldova [Internet]. Copenhagen; 2014. Available from: http://www.euro.who.int/__data/assets/pdf_file/0008/255464/BetterNCDoutcomesChallengesOpportunitiesHealthSystemsMoldovaCountryAssessmentEng.pdf?ua=1
14. Jackson R, Ameratunga S, Broad J, Connor J, Lethaby A, Robb G, et al. The GATE frame: critical appraisal with pictures. Vol. 144, ACP journal club. United States; 2006. p. A8-11.
15. Gale NK, Heath G, Cameron E, Rashid S, Redwood S. Using the framework method for the analysis of qualitative data in multi-disciplinary health research. BMC Med Res Methodol. 2013 Sep;13:117.

For peer review only

For peer review only - http://bmjopen.bmj.com/site/about/guidelines.xhtml

AUTHORS AND CONTRIBUTIONS

DC, AC, TL, GC, VS, TZ, AA, and JF contributed to the methodological design. DC, AC, TL, and JF contributed to writing the manuscript.

FUNDING STATEMENT

This study is funded jointly by the Swiss Agency for Development and Cooperation (SDC) and WHO Regional Office for Europe.

COMPETING INTERESTS STATEMENT

DC, AC, TL, GC, VS, TZ, AA, JF declare no competing interests.

For peer review only

443 **Table 1.** Indicators, their numerators and denominators, and questions the indicators answer

Question	Indicator	Numerator	Denominator
Are risk factors being measured?	Proportion of eligible patients who have all risk factor values recorded as required for calculation of risk score	Patients aged 40 or older who have visited in the last 12 months who have all measurements required for calculation of risk score within 12 months of the most recent date of visit	Patients aged 40 or older who have visited in the last 12 months
Are risk factor measurements being converted to a total risk score?	Proportion of patients aged 40 or older who have visited in the last 12 months who have all measurements required for calculation of risk score within 12 months of the most recent date of visit, that have a documented risk score	Patients aged 40 or older who have visited in the last 12 months who have all measurements required for calculation of risk score within 12 months of the most recent date of visit, that have a documented risk score	Patients aged 40 or older who have visited in the last 12 months who have all measurements required for calculation of risk score within 12 months of the most recent date of visit
Are risk scores calculated correctly?	Proportion of patients aged 40 or older who have visited in the last 12 months who have all measurements required for calculation of risk score within 12 months of the most recent date of visit, that have a documented risk score that is correct	Patients aged 40 or older who have visited in the last 12 months who have all measurements required for calculation of risk score within 12 months of the most recent date of visit, that have a documented risk score that is correct	Patients aged 40 or older who have visited in the last 12 months who have all measurements required for calculation of risk score within 12 months of the most recent date of visit, that have a documented risk score
Are patients being risk scored?	Proportion of eligible patients with a documented risk score	Patients aged 40 or older who have visited in the last 12 months with a documented risk score	Patients aged 40 or older who have visited in the last 12 months
Are risk scores calculated correctly?	Proportion of eligible patients with a documented risk score that is correct	Patients aged 40 or older who have visited in the last 12 months with a documented risk score that is correct	Patients aged 40 or older who have visited in the last 12 months with a documented risk score
Are statins prescribed to the correct patients?	Proportion of eligible patients prescribed a statin	Patients with existing CVD, diabetics 40 or older with high LDL values (as defined based on total CVD risk of SCORE 10-14% in LDL ≥2.6 mmol/L; with very high risk SCORE ≥15% in LDL ≥1.8 mmol/L), or patients with a SCORE of ≤ 9% and LDL ≥ 2.6 or total cholesterol ≥7.2, or patients with a SCORE of 10-14% and a LDL >=1.8 or total cholesterol ≥7.2 mmol/L, or patients with a SCORE of ≥15%, prescribed a statin	Patients with existing CVD, diabetics 40 or older with high LDL values (as defined based on total CVD risk of SCORE 10-14% in LDL ≥2.6 mmol/L; with very high risk SCORE ≥15% in LDL ≥1.8 mmol/L), or patients with a SCORE of ≤ 9% and LDL ≥2.6 or total cholesterol ≥ 7.2, or patients with a SCORE of 10-14% and a LDL ≥1.8 or total cholesterol ≥7.2 mmol/L, or patients with a SCORE of ≥15%
Are statins prescribed correctly based on documented risk score?	Proportion of patients eligible based on documented risk score prescribed a statin	Patients with a documented risk score as very high risk SCORE ≥15% prescribed a statin	Patients with a documented risk score as very high risk SCORE ≥15%
Are patients with existing disease, who do not require the calculation of a risk score to prescribe satins, prescribed statins?	Proportion of patients with existing CVD prescribed a statin	Patients with existing CVD prescribed a statin	Patients with existing CVD

For peer review only - http://bmjopen.bmj.com/site/about/guidelines.xhtml

Is the blood pressure of high risk patients controlled?	Proportion of high risk patients (SCORE ≥15% or DM and age over 40) whose last two recorded blood pressure measurements were <130/80 mmHg	Patients with a true risk score indicating a very high risk (SCORE ≥15%) or DM and age over 40 whose last two documented blood pressure readings were <130/80	Patients with a true risk score indicating a very high risk (SCORE ≥15%) or DM and age over 40
Is the blood pressure of lower risk patients controlled?	Proportion of lower risk patients (SCORE<15%) whose last two recorded blood pressure measurements were <140/90 mmHg	Patients with a true risk score indicating <15% whose last two documented blood pressure readings were <140/90	Patients with a true risk score indicating <15%
Are patients with existing CVD prescribed basic medications to reduce risk?	Proportion of patients with existing CVD prescribed a statin and aspirin and blood pressure lowering treatment	Patients with existing CVD prescribed a statin and aspirin and blood pressure lowering treatment	Patients with existing CVD
Is the blood glucose of diabetic patients controlled?	Proportion of diabetic patients with glycaemic control as defined by last two HbA1c measurements	Patients with diabetes 2 whose last two HbA1c measurements were below personal target as defined by MDA adapted WHO PEN 1	Patients with diabetes type 2
Is the blood pressure of hypertensive patients controlled?	Proportion of confirmed hypertensive patients whose SBP is <140/90 at last two visits	Patients with confirmed hypertension whose last two blood pressure readings were <140/90	Patients with confirmed hypertension
What is the prevalence of high blood pressure?	Proportion of people whose last two systolic blood pressure reading are 140 mmHg or above	Patients whose last two systolic blood pressure readings were ≥140	All patients over 18

For peer review only

For peer review only - http://bmjopen.bmj.com/site/about/guidelines.xhtml

Table 2. Standardized data collection form used to extract data from individual patient records

**Data Collection Question**	**Answer**
What is your name? (Name of person extracting data)	
Date of Data Extraction (MM-DD-YYYY)	
Write the Clinic Name	
Is this a duplicate extraction?	
If it is a duplicate extraction, enter the number you and your extraction partner have assigned to this file.	
Date of Birth (MM-DD-YYYY)	
Sex (M/F)	
Smoking Status (Y/M)	
Diagnosis of Hypertension (Y/N)	
Date of Hypertension Diagnosis (MM-DD-YYYY)	
Can you find one or more blood pressure readings? (Y/N)	
Most Recent Systolic Blood Pressure	
Most Recent Diastolic Blood Pressure	
Date of the Most Recent Blood Pressure Measurement (MM-DD-YYYY)	
Can you find a second most recent blood pressure reading? (Y/N)	
Second most recent systolic blood pressure	
Second most recent diastolic blood pressure	
Date of the second most recent systolic blood pressure (MM-DD-YYYY)	
Diagnosis of Diabetes (Type 1, Type 2, No)	
Can you find one or more HbA1c measurements? (Y/N)	
Most recent HbA1c reading (mmol/mol)	
Date of the most recent HbA1c measurement? (MM-DD-YYYY)	
Can you find another HbA1c measurement? (Y/N)	
Second most recent HbA1c reading (mmol/mol, otherwise specify unit)	
Date of the second most recent HbA1c reading? (MM-DD-YYYY)	
Can you find one or more total cholesterol measurements? (Y/N)	
Most recent total cholesterol reading (mmol/L)	
Date of the most recent cholesterol reading (MM-DD-YYYY)	
Can you find another cholesterol measurement? (Y/N)	
Second most recent cholesterol reading (mmol/L)	
Date of the second most recent cholesterol reading (MM-DD-YYYY)	
Was the patient prescribed a statin? (Y/N)	
What was the date of the statin prescription? (MM-DD-YYYY)	

For peer review only

For peer review only - http://bmjopen.bmj.com/site/about/guidelines.xhtml

What was the drug and dose?	
Does the patient have existing CVD? (Y/N)	
State the type of CVD	
Has the patient been prescribed acetylsalicylic acid (ASA or aspirin)? (Y/N)	
What was the most recent date that ASA was prescribed? (MM-DD-YYYY)	
Has the patient been prescribed anti-hypertensives? (Y/N)	
What was the most recent date that anti-hypertensives were prescribed? (MM-DD-YYYY)	
Can you find a documented ESC SCORE risk score? (Y/N)	
Enter the most recent documented ESC SCORE risk score (%)	
What was the date the risk score was documented? (MM-DD-YYYY)	
Please record any important notes about the data extraction here. Examples include an error you think may have been made, clarification of the units for measurements (e.g. mmol/L vs mg/dL). Or notes that you would like for yourself.	

449

For peer review only

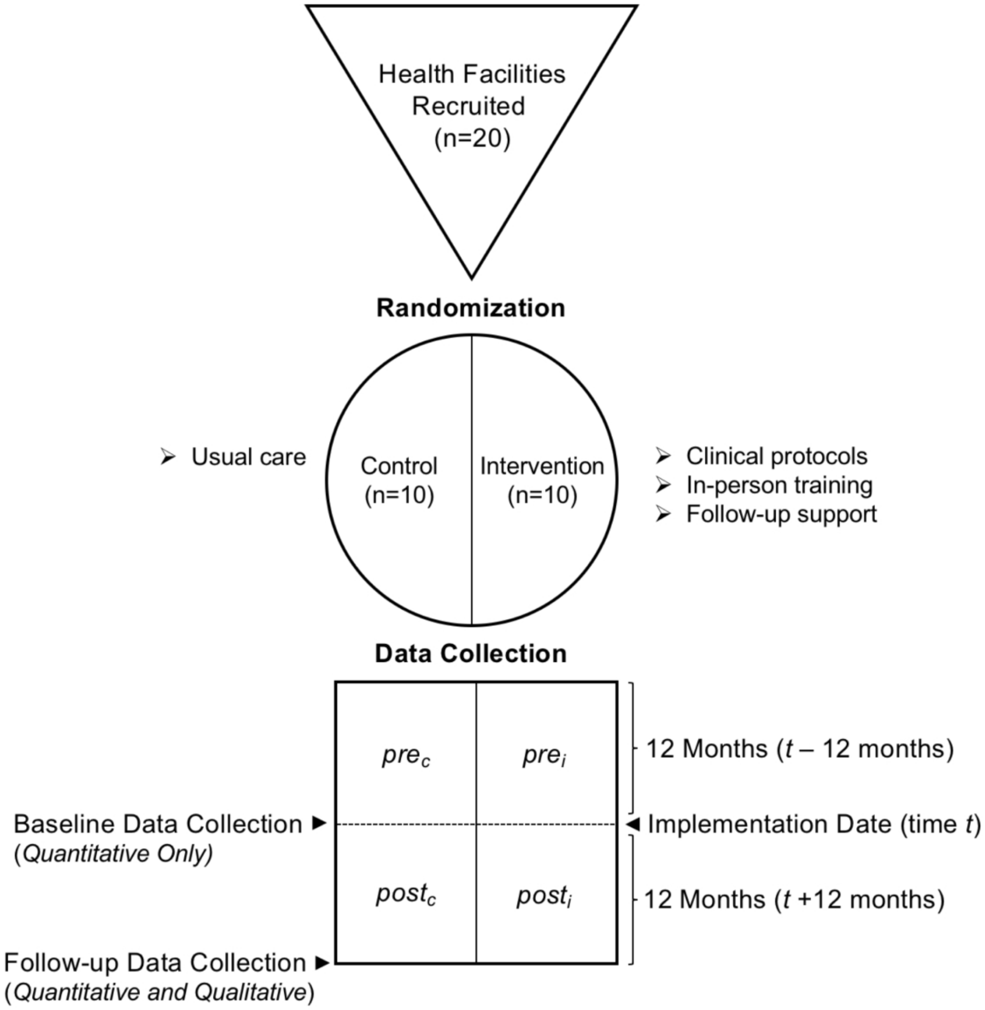

Figure 1. Illustration using the GATE frame structure (14) of the mixed methods evaluation design

184x193mm (300 x 300 DPI)

Protocol for the Evaluation of a Pilot Implementation of Essential Interventions for the Prevention of Cardiovascular Diseases in Primary Health Care in the Republic of Moldova

Journal:	*BMJ Open*
Manuscript ID	bmjopen-2018-025705.R2
Article Type:	Protocol
Date Submitted by the Author:	02-Jun-2019
Complete List of Authors:	Collins, Dylan; University of British Columbia,<br>Ciobanu, Angela<br>Laatikainen, Tiina; National Institute for Health and Welfare, Epidemiology and Health Promotion<br>Curocichin, Ghenadie<br>Salaru, Virginia<br>Zatic, Tatiana<br>Anisei, Angela<br>Farrington, Jill
**Primary Subject Heading**:	Cardiovascular medicine
Secondary Subject Heading:	Public health
Keywords:	Cardiology < INTERNAL MEDICINE, PRIMARY CARE, Quality in health care < HEALTH SERVICES ADMINISTRATION & MANAGEMENT, Protocols & guidelines < HEALTH SERVICES ADMINISTRATION & MANAGEMENT

Protocol for the Evaluation of a Pilot Implementation of Essential Interventions for the Prevention of Cardiovascular Diseases in Primary Health Care in the Republic of Moldova

Dylan R. J. Collins[1], Angela Ciobanu[2], Tiina Laatikainen[3], Ghenadie Curocichin[4], Virginia Salaru[4], Tatiana Zatic[5], Angela Anisei[6], Jill L. Farrington[7]

[1]University of British Columbia, Vancouver, Canada
[2]World Health Organization Country Office in Republic of Moldova, Chisinau, Republic of Moldova
[3]Institute of Public Health and Clinical Nutrition, Helsinki, Finland
[4]Family Medicine Department, Nicolae Testemitanu State Medical and Pharmaceutical University, Chisinau, Republic of Moldova
[5]Primary, Emergency and Community Health Policies Department, Ministry of Health, Labour and Social Protection, Chisinau, Republic of Moldova
[6]Department on Quality Management of Health Services, National Public Health Agency, Republic of Moldova
[7]World Health Organization Regional Office for Europe, Copenhagen, Denmark

Correspondence to Dr. Dylan Collins at dylan.collins@alumni.ubc.ca

For peer review only

ABSTRACT

Introduction

Nearly 90% of all deaths in Republic of Moldova are caused by NCDs, the majority of which (55%) are caused by CVD. In addition to reducing premature mortality from CVD, it is estimated that strengthening primary health care could cut the number hypertension-related hospital admissions and diabetes-related hospitalizations in half. The aim of this evaluation is to determine the feasibility of implementing and evaluating essential interventions for the prevention of CVD in primary health care in Republic of Moldova, with a view toward national scale-up.

Methods and Analysis

A national steering group including international experts will be convened to adapt WHO PEN protocols one and two to the health system of Republic of Moldova, develop and conduct training of primary health care workers, and test a core set of indicators to monitor the quality of care and change in clinical practice. To evaluate the impact of this pilot implementation, a pragmatic, sequential mixed methods explanatory design, composed of quantitative and qualitative strands of equal weight, will be used. Twenty primary health care centres will be selected and randomized to the training and implementation arm (n=10) and the usual care arm (n=10). At baseline and 12 months follow-up, a standardized data collection form will be piloted to extract data directly from patient paper records in order to estimate the change in clinical practice. Semi-structured interviews and inter-clinic peer workshops will be conducted at 12 months follow-up, and qualitative data collected from these formats will be analysed thematically for explanatory themes that relate to the quantitative findings.

Ethics and Dissemination

Ethical review and approval has been obtained. Findings of the evaluation will be shared in a project report to key stakeholders, presented back to participants, and written into a manuscript for an open access peer-reviewed scientific journal.

STRENGTHS AND LIMITATIONS OF THIS STUDY

- To our knowledge, this is the first description of adapting and piloting WHO essential NCD interventions in primary health care in a low- or middle-income country and provides a methodological example to other jurisdictions
- A mixed methods design allows for a greater understanding of the potential barriers and facilitators to implementation and can inform future health systems development
- Primary health care facilities will be selected from different regions of Republic of Moldova in order to pilot implementation in a variety of contexts throughout the country
- Since this is an evaluation of a pilot implementation, the sample size is based on pragmatism and not statistical power
- We are unable to include patient perspectives and experience in the evaluation, which is an important aspect of health care quality

For peer review only - http://bmjopen.bmj.com/site/about/guidelines.xhtml

INTRODUCTION

Globally, non-communicable diseases (NCDs) account for more than one-half of the global burden of disease.(1) In 2016, an estimated 41 million deaths were due to NCDs, of which nearly half were due to cardiovascular diseases (CVD).(2) Primary health care systems play an important role in the prevention, early detection, and appropriate management of these diseases, but many nations lack primary health care capacity.(3,4)

To support national governments to realize their commitments in reducing the burden of NCDs, as agreed in the United Nations Political Declaration on NCDs, the World Health Assembly endorsed the WHO Global Action Plan for the Prevention and Control of NCDs 2013-2020. To support implementation of this Action Plan, WHO has identified a set of cost-effective policy options ("best buys") for the prevention and control of NCDs within countries.(5)

The Republic of Moldova (henceforth "MDA") is located in Eastern Europe, between Ukraine and Romania; the Capital and largest city is Chisinau. By gross domestic product per capita, MDA is one of the poorest countries in the WHO European Region and it is estimated that 21.9% of citizens live below the absolute poverty line of 1 US Dollar per day.(6)

Non-communicable diseases are a leading cause of death in MDA

While NCDs are a global epidemic, MDA ranks amongst the countries most affected. Nearly 90% of all deaths in MDA are caused by NCDs, the majority of which (55%) are caused by CVD.(7) In 2016, the probability of dying prematurely from any of the four major NCDs (CVDs, cancer, diabetes, chronic respiratory disease) was 24.9%,; almost twice as high for men (33.7%) as women (17.3%).(8) Men and people residing in rural areas are disproportionally impacted by CVD and represent key populations for public health intervention.(7)

This burden is driven by some of the highest rates of NCD risk factors, including tobacco and alcohol use, in the WHO European region indicated by a 2013 STEPS survey.(9) One-in-four (25.3%) Moldovans smoke tobacco and this rate nearly doubles in men.(9) Among adults aged 18 to 69, 61.9% currently consume alcohol and one in five people have engaged in heavy episodic drinking (six or more drinks on any one occasion in the past 30 days).(9)

The overall prevalence of obesity amongst adults is 22.9%, being higher among women (28.5%) as compared with men (17.8%).(9) The prevalence of raised blood pressure (defined as SBP $\geq$ 140 mmHg and/or DBP $\geq$ 90 mmHg or currently taking medication for raised blood pressure) among MDA's adult population is 39.8%, and 76.2% of these patients are not on blood pressure lowering medication.(9) A total of 12.3% of the population have a blood glucose level of $\geq$ 6.1 mmol/L, and 29.4% of the population has a total blood cholesterol level of $\geq$ 5 mmol/L.(9) It is estimated that one in five (23.0%) people aged 40–69 years have a 10-year fatal or non-fatal CVD risk of over 30% (including those with an existing CVD).(9)

Primary health care in MDA and commitment to NCDs

According to the Constitution of Republic of Moldova of 1994, citizens are entitled to a free of charge minimum package of essential health services, including primary health care. However, resource constraints have made it difficult to offer these services and significant gaps in care

For peer review only - http://bmjopen.bmj.com/site/about/guidelines.xhtml

exist.(10) According to the most recent data (2010), there were 5.3 family doctors per 10,000 inhabitants and 25.9 specialist doctors per 10,000 inhabitants. In rural areas these rates are halved, leading to human resource shortages in primary care.(10) Approximately 17% of practicing physicians in MDA work in primary health care, and 92% of them rely on paper clinical records.(6) The most recent estimate (2009) states that there are approximately 630 primary health care centres throughout the country, or 21.2 centres per 100,000 people.(6)

Despite these health system challenges, the Government of Republic of Moldova is committed to improving primary health care capacity for NCDs. It is estimated that 60% of hypertension-related hospital admissions (about 12,000 annually) and 40% of diabetes-related hospitalizations (about 5,000 annually) could be prevented through strengthened primary health care for these conditions, including better identification and management of those at increased CVD risk.(11)

Given the need and international policy support for addressing this gap in NCD care, there was a favourable window of opportunity to act with impact. As such, strengthening primary health care was set out as one of the main commitments in the Action Program of the Government of Republic of Moldova 2016–2018.(12) To do this requires the development of simplified clinical protocols, in-person training programs for nurses and doctors, and a core set of indicators to monitor and evaluate changes in the quality of care.

Essential interventions to prevent cardiovascular diseases in primary health care

In order to build capacity in primary health care and ultimately prevent premature mortality from CVD in MDA, a study was envisioned to adapt and pilot the World Health Organization Package of Essential NCD Intervention from Primary Healthcare in Low Resource Settings (WHO PEN).(3) WHO PEN includes simplified clinical protocols which together cover the integrated management of hypertension and diabetes, as well as education and counselling on healthy behaviours aimed to prevent CVD. The central strategy of this integrated approach is the use of total cardiovascular risk assessment to stratify and target individuals at high CVD risk, a process considered to be a “best buy” intervention by the WHO.(5)

These interventions are aimed at tackling areas identified in a 2014 WHO assessment of challenges and opportunities for better NCD outcomes in Moldova. (13) This includes shortcomings amongst health workers in the identification and management of individuals with increased cardiovascular risk. The interventions are expected to add to the current quality of care by targeting interventions (non-pharmacological and/or pharmacological) to those at highest risk who stand to gain the most in absolute cardiovascular risk reduction, while also emphasizing improvements in the organization of care. The intervention also includes practical face-to-face training and follow-up implementation support. Current practice underutilizes these medical strategies and guidelines (e.g. CVD risk score directed primary prevention), in addition to limited task sharing with non-physician health works (e.g. nurses) in these care pathways. (13) At the study’s inception, there were no known developments beyond the scope of this project that could change clinical practice for NCDs in primary health care.

Since the use of WHO PEN was unprecedented in MDA, the Ministry of Health, Labour and Social Protection convened a national steering group to lead the adaptation and pilot process, with the goal of using the findings for future health systems development. Led by the primary health care

For peer review only - http://bmjopen.bmj.com/site/about/guidelines.xhtml

division of the Ministry of Health, the steering group is comprised of representatives from the Nicolae Testemitanu State University of Medicine and Pharmacy and the National Public Health Agency. The national steering group is supported by an international team of experts coordinated jointly by the WHO Regional Office for Europe and WHO Country Office in the Republic of Moldova.

AIM AND OBJECTIVES

Aim

The aim of the evaluation is to determine the feasibility of implementing and evaluating essential interventions for the prevention of cardiovascular disease in primary health care in MDA, with a view toward national scale-up.

Objectives

Primary Objectives

1. Assess the ability to implement MDA-adapted WHO PEN protocols one and two in pilot primary health care centres
2. Determine the feasibility of collecting quantitative data required for future studies of effectiveness from the existing informal paper clinical record system

Secondary Objectives

1. Determine the baseline performance of primary health care services with respect to essential interventions for the prevention and management of CVD
2. Estimate the change in performance of pilot primary health care centres after 12 months of protocol implementation and compare this to control clinics using usual care

METHODS AND ANALYSIS

Overview of Process and Design

An overview of the methods used to adapt, pilot, and evaluate essential interventions for CVD in primary health care in MDA are summarized by the following seven steps, which are planned to occur from September 2016 to May 2019.

Step One: Adaptation of WHO PEN Protocols to the National Context

Under the direction of the national steering group, WHO PEN protocols one and two will be compared and contrasted to national disease specific guidelines. The WHO PEN protocols will then be adapted to ensure consistency with the organization, culture, and availability of resources of the health system, while ensuring that they remain simple clinical decision support tools.

Step Two: Development of a Training Package for Primary Health Care Workers

A three-day training package will be developed under the direction of the national steering group in order to provide in-person theoretical and practical training to nurses and doctors working in primary health care. This will include lectures, clinical case studies, and practical exercises that embrace the experience and knowledge of participants.

Step Three: Collection of Baseline Data

For peer review only - http://bmjopen.bmj.com/site/about/guidelines.xhtml

According to the Ministry of Health process, a list of 20 primary health care clinics will be nominated and provided to the working group. They will then be randomized into an intervention group arm (n=10) and control arm (n=10). Data for quantitative indicators will be extracted from all 20 clinics by randomly sampling individual paper-based patient records from all primary health care units using a standardized data collection instrument. This will be done before randomization by a specially trained group of postgraduate medical trainees, such that neither the clinics nor the data extractors will know the allocation of each clinic to intervention or control arm.

Step Four: Training Staff in Pilot Clinics

All doctors and nurses from the primary health care centres in the intervention arm will be invited to be trained together by a national team of experts in groups of approximately 30. It is estimated that up to 200 health workers will be trained in total. At the end of training each PHC team will pass through evaluation at the University Centre for Simulation in Medical Training using objective structured clinical exams and get feedback from trainers and peers.

Step Five: Implementation of Protocols

Trained participants from the ten primary health care clinics in the intervention arm will then be free to implement the clinical protocols and change their clinical practice, without incentives, for 12 months. During this time, a team of national experts will be created to offer support (distance and on-the-job) to the primary health care centres in the intervention arm. All ten clinics in the intervention arm will receive at least one in-person follow-up support visit.

Step Six: Collection of Follow-up Data

After 12 months, using the same method and data collection instruments used to collect baseline quantitative data (Step Three), data will again be extracted from randomly selected individual paper-based patient records from all 20 health care centres. Five primary health care centres from the intervention arm will be selected by the national steering group for one-on-one semi structured interviews with health staff. This will be supplemented by inviting a selection of staff from all ten health centres in the intervention arm to participate in focus groups. Together, these qualitative data will be analysed thematically for explanatory themes.

Step Seven: Evaluation of Results and Sharing Experience

The findings of the quantitative and qualitative analyses will be integrated in a final report and shared with key stakeholders, including health staff from the participating primary health care centres. The results will also be shared at a national conference and in an open-access peer reviewed journal, in order to inform the future development of primary health care capacity in MDA.

Methodological Design

A pragmatic, sequential mixed methods explanatory design, composed of quantitative and qualitative strands of equal weight, will be used (Figure 1). This design was chosen because it allows for the use of qualitative data to enlighten and explain the quantitative findings, including but not limited to the feasibility of collecting data from paper-based records, the contextual factors affecting guideline implementation, changes in clinical practice, and optimization for the future.

Figure 1. Illustration using the GATE frame structure (14) of the mixed methods evaluation design

For peer review only - http://bmjopen.bmj.com/site/about/guidelines.xhtml

A sample size of 20 primary health care centres was chosen because it was seen as a good balance of allowing for variation in clinic geography and demography, while still remaining feasible for the pilot implementation. Half of the centres (n=10) will be randomly allocated to the intervention arm and half (n=10) to the control arm. Baseline data will be collected from both intervention and control clinics, ensuring that baseline data is collected before implementation occurs.

Within clinic comparisons will be used to compare the 12 months before randomization with the 12 months of implementation. Between clinics comparison will be used to compare the intervention clinics with control clinics during the same time period.

Eligibility Criteria for Primary Health Care Centres

Health facilities will be nominated by the Ministry of Health for participation based on the following eligibility criteria: (1) primary health care facilities must be operating in the public sector as legal entities; (2) primary health care facilities must be sampled in a way such that they are geographically distributed evenly across the country; equally from the Central, North and Southern regions of MDA; and (3) health facilities must be primary health care centres that are managed by family doctors with no specialist doctors working in the facility. These criteria were chosen in order to select a group of clinics that sufficiently reflect the majority of primary health care facilities in Moldova.

Randomization

The clinics will be stratified based on the ratio of patients to family doctors to minimize possible confounding by doctor caseload, and then randomized electronically into two groups of 10 primary health care centres.

Comparison

The 10 primary health care centres in the intervention arm will be compared to the 10 primary health care centres in the control arm. The control arm will receive no intervention and proceed with usual care.

Quantitative Indictors

Indicators were developed to balance input and process indicators, such as measurement of risk factors and calculation of risk scores, with output (e.g. prescribing) and outcome (e.g. blood pressure control) indicators. While one of the objectives of this evaluation is to determine the ability to measure these indicators based on routine paper records, we used our existing knowledge of the health system to design indicators which were valuable and likely to be feasible to calculate. Table 1 shows the indicator, the question the indicator seeks to answer, and the respective numerator and denominator definitions which will be used in the calculations.

Table 1. Indicators, their numerators and denominators, and questions the indicators answer

Data Collection and Management

Quantitative Data Collection Tool

For peer review only

For peer review only - http://bmjopen.bmj.com/site/about/guidelines.xhtml

A standardized data collection template has been developed for extracting anonymized patient data from individual paper records (Table 2). An online version was also made to allow for data entry on a computer or smartphone. It is estimated to take 15 minutes to extract data from one patient record since the records are made of blank paper with no formal structure or organization of health data.

Table 2. Standardized data collection form used to extract data from individual patient records

Method of Randomly Sampling Patient Records

A random sample of the records of patients aged over 18, who have visited the medical facility within the past 12 months, will be taken. Since medical records in MDA are organized alphabetically on shelves, we created a randomly generated list of alphanumeric combinations that allowed for the selection of patient charts at random. For example, an alphanumeric code of "C24" would correspond to the 24th patient chart in the section of last names starting with the letter C.

The list will be followed in the order that it was generated so as to prevent selection bias. The randomly selected chart will then be checked to see if it meets two inclusion criteria: (1) the patient is aged 18 years or older and (2) the patient visited the health centre within the last 12 months. If the record meets these criteria, data will then be extracted. If it does not, it will be returned to the shelf and the next alphanumeric code on the randomly generated list will be used. This process will be repeated in each clinic until a sample size of 1.2% of the patient population in each clinic is sampled. This proportion was chosen pragmatically such that the average sample per primary health care centre would equal 100 unique patients.

Data Analysis

The change in indicators from baseline to follow-up will be calculated for intervention clinics and compared with control clinics (Table 1). Subgroup analysis by age, gender, and other demographic features may be done as deemed appropriate by the national steering committee. All analyses will account for stratified sampling. Since the health centre is the unit of inference for the outcomes (e.g. health centre proportion of eligible patients with a documented CVD risk score), use of an intracluster correlation coefficient is not required for analyses of these outcomes. Age and gender adjusted logistic regression models will be used to analyse the differences in pre-defined indicators between intervention and control clinics and between baseline and follow-up. The differences in means of continuous variables between the intervention and control clinics and baseline and follow-up will be analysed using age and gender adjusted analysis of variance.

Qualitative Data Collection

Follow-up Support Visits

Follow-up visits will be made to each intervention clinic at least once during the implementation timeframe (12 months) to provide ad hoc implementation support. These visits will be conducted by members of the national steering group, who will keep field notes about each visit and provide feedback and support to the health centres. The perspectives gained through follow-up support visits will be used by the national steering group to develop preliminary data collection tools for semi-structured interviews.

For peer review only

For peer review only - http://bmjopen.bmj.com/site/about/guidelines.xhtml

Semi-Structured Interviews

A maximum variation sample of half of the intervention clinics (n=5) will be chosen, based on the perceived performance of each clinic by the evaluation steering committee. A pragmatic sample of clinic managers (n=1 per clinic), doctors (n=3 per clinic), and nurses (n=3 per clinic) will be interviewed one-on-one, using a semi-structured format. Interviews will proceed until data saturation has been reached, to a maximum of 30 interviews. After obtaining written, informed consent, interviews will be of 30 to 60 minutes in length, audio recorded, and be transcribed verbatim and analysed thematically using framework thematic analysis.(15) The interviews will be conducted by members of the steering group, but the interviewers will be allocated to participants from health centres with whom they did not provide follow-up support visits.

Focus Group Workshop

Participants from all ten implementation clinics will be invited to a workshop to further collect explanatory qualitative data and to critically reflect on the implementation process. Participants will be a mix of doctors, nurses, and managers from the intervention clinics.

Participants will be placed into small groups based on their profession, and asked to complete a standardized worksheet. Each group will be under the guidance of a facilitator, and emergent themes from one-one-one interviews will be used as prompts to each group. The worksheet will allow for each group to directly comment, modify, or add to the emergent themes, create new themes, and organize themes into categories such as barriers and facilitators.

<u>Integration of Quantitative and Qualitative Strands</u>

The resulting qualitative data will be analysed thematically using the framework approach, and used to help explain the findings of the quantitative strand.(15) Following the sequential mixed method design, integration of the qualitative findings with quantitative findings will allow for the interpretation of the results in light of each other. This may include post-hoc analysis of effectiveness of some of the quantitative outcomes as appropriate, to further add meaning to the integration of qualitative and quantitative strands.

Patient and Public Involvement

Neither patients nor the public were involved in the methodological design.

ETHICS AND DISSEMINATION

Ethical Review and Approval

This project was reviewed by the Research Ethics Committee of the Nicolae Testemitanu State University of Medicine and Pharmacy of the Republic of Moldova and granted permission on 31 May 2017.

Dissemination

Quantitative findings will be summarized and presented back to all intervention clinics during follow-up workshops. A comprehensive project report will be written and shared with key stakeholders. A final report of key findings of the evaluation will be written and submitted to an open access peer-reviewed journal and made available to all study participants so they can use the findings to improve their practice. The findings will be used to evaluate the feasibility of a national scale-up of essential NCD interventions in primary health care in MDA.

For peer review only - http://bmjopen.bmj.com/site/about/guidelines.xhtml

REFERENCES

1. Benziger CP, Roth GA, Moran AE. The Global Burden of Disease Study and the Preventable Burden of NCD. Glob Heart. 2016 Dec;11(4):393–7.
2. World Health Organization (WHO). World Health Statistics [Internet]. 2018. Available from: http://www.who.int/gho/publications/world_health_statistics/2018/en/
3. World Health Organization (WHO). Package of Essential Noncommunicable (PEN) disease interventions for primary health care in low-resource settings [Internet]. 2013. Available from: http://www.who.int/ncds/management/pen_tools/en/
4. World Health Organization (WHO). Global action plan for the prevention and control of NCDs 2013-2020. 2013.
5. Organization WH. Tackling NCDs: "Best buys" and other recommended interventions for the prevention and control of noncommunicable diseases [Internet]. 2017. Available from: http://www.who.int/ncds/management/best-buys/en/
6. World Health Organization (WHO). Evaluation of the sturcutre and provision of pirmary care in the Republic of Moldova. 2012.
7. National Center for Health Management. Health Yearbook: Public Health in Moldova [Internet]. 2015. Available from: http://www.cnms.md/ro/rapoarteo
8. World Health Organization. Global Health Observatory data repository [Internet]. [cited 2018 Jul 10]. Available from: http://apps.who.int/gho/data/node.home
9. World Health Organization (WHO). Prevalence of Noncommunicable Disease Risk Factors in the Republic of Moldova (STEPS) [Internet]. 2014. Available from: http://www.who.int/ncds/surveillance/steps/Moldova_2013_STEPS_Report.pdf
10. Turcanu C, Domente S, Buga M, Richardson E. Health Systems in Transition: Republic of Moldova [Internet]. 2012. Available from: http://www.euro.who.int/__data/assets/pdf_file/0006/178053/HiT-Moldova.pdf
11. World Health Organization (WHO). Ambulatory care sensitive conditions in the Republic of Moldova [Internet]. 2015. Available from: http://www.euro.who.int/en/countries/republic-of-moldova/publications/ambulatory-care-sensitive-conditions-in-the-republic-of-moldova-2015
12. Government of the Republic of Moldova. Action Program of the Government of the Republic of Moldova 2016–2018 [Internet]. 2016. Available from: http://gov.md/sites/default/files/document/attachments/guvernul_republicii_moldova_-_programul_de_activitate_al_guvernului_republicii_moldova_2016-2018.pdf
13. World Health Organization Regional Office for Europe. Better noncommunicable disease outcomes: challenges and opportunities for health systems -- Country Assessment of Republic of Moldova [Internet]. Copenhagen; 2014. Available from: http://www.euro.who.int/__data/assets/pdf_file/0008/255464/BetterNCDoutcomesChallengesOpportunitiesHealthSystemsMoldovaCountryAssessmentEng.pdf?ua=1
14. Jackson R, Ameratunga S, Broad J, Connor J, Lethaby A, Robb G, et al. The GATE frame: critical appraisal with pictures. Vol. 144, ACP journal club. United States; 2006. p. A8-11.
15. Gale NK, Heath G, Cameron E, Rashid S, Redwood S. Using the framework method for the analysis of qualitative data in multi-disciplinary health research. BMC Med Res Methodol. 2013 Sep;13:117.

For peer review only - http://bmjopen.bmj.com/site/about/guidelines.xhtml

AUTHORS AND CONTRIBUTIONS

DC, AC, TL, GC, VS, TZ, AA, and JF contributed to the methodological design. DC, AC, TL, and JF contributed to writing the manuscript.

FUNDING STATEMENT

This study is funded jointly by the Swiss Agency for Development and Cooperation (SDC) and WHO Regional Office for Europe.

COMPETING INTERESTS STATEMENT

DC, AC, TL, GC, VS, TZ, AA, JF declare no competing interests.

For peer review only

443 **Table 1.** Indicators, their numerators and denominators, and questions the indicators answer

Question	Indicator	Numerator	Denominator
Are risk factors being measured?	Proportion of eligible patients who have all risk factor values recorded as required for calculation of risk score	Patients aged 40 or older who have visited in the last 12 months who have all measurements required for calculation of risk score within 12 months of the most recent date of visit	Patients aged 40 or older who have visited in the last 12 months
Are risk factor measurements being converted to a total risk score?	Proportion of patients aged 40 or older who have visited in the last 12 months who have all measurements required for calculation of risk score within 12 months of the most recent date of visit, that have a documented risk score	Patients aged 40 or older who have visited in the last 12 months who have all measurements required for calculation of risk score within 12 months of the most recent date of visit, that have a documented risk score	Patients aged 40 or older who have visited in the last 12 months who have all measurements required for calculation of risk score within 12 months of the most recent date of visit
Are risk scores calculated correctly?	Proportion of patients aged 40 or older who have visited in the last 12 months who have all measurements required for calculation of risk score within 12 months of the most recent date of visit, that have a documented risk score that is correct	Patients aged 40 or older who have visited in the last 12 months who have all measurements required for calculation of risk score within 12 months of the most recent date of visit, that have a documented risk score that is correct	Patients aged 40 or older who have visited in the last 12 months who have all measurements required for calculation of risk score within 12 months of the most recent date of visit, that have a documented risk score
Are patients being risk scored?	Proportion of eligible patients with a documented risk score	Patients aged 40 or older who have visited in the last 12 months with a documented risk score	Patients aged 40 or older who have visited in the last 12 months
Are risk scores calculated correctly?	Proportion of eligible patients with a documented risk score that is correct	Patients aged 40 or older who have visited in the last 12 months with a documented risk score that is correct	Patients aged 40 or older who have visited in the last 12 months with a documented risk score
Are statins prescribed to the correct patients?	Proportion of eligible patients prescribed a statin	Patients with existing CVD, diabetics 40 or older with high LDL values (as defined based on total CVD risk of SCORE 10-14% in LDL ≥2.6 mmol/L; with very high risk SCORE ≥15% in LDL ≥1.8 mmol/L), or patients with a SCORE of ≤ 9% and LDL ≥ 2.6 or total cholesterol ≥7.2, or patients with a SCORE of 10-14% and a LDL >=1.8 or total cholesterol ≥7.2 mmol/L, or patients with a SCORE of ≥15%, prescribed a statin	Patients with existing CVD, diabetics 40 or older with high LDL values (as defined based on total CVD risk of SCORE 10-14% in LDL ≥2.6 mmol/L; with very high risk SCORE ≥15% in LDL ≥1.8 mmol/L), or patients with a SCORE of ≤ 9% and LDL ≥2.6 or total cholesterol ≥ 7.2, or patients with a SCORE of 10-14% and a LDL ≥1.8 or total cholesterol ≥7.2 mmol/L, or patients with a SCORE of ≥15%
Are statins prescribed correctly based on documented risk score?	Proportion of patients eligible based on documented risk score prescribed a statin	Patients with a documented risk score as very high risk SCORE ≥15% prescribed a statin	Patients with a documented risk score as very high risk SCORE ≥15%
Are patients with existing disease, who do not require the calculation of a risk score to prescribe satins, prescribed statins?	Proportion of patients with existing CVD prescribed a statin	Patients with existing CVD prescribed a statin	Patients with existing CVD

For peer review only - http://bmjopen.bmj.com/site/about/guidelines.xhtml

Is the blood pressure of high risk patients controlled?	Proportion of high risk patients (SCORE ≥15% or DM and age over 40) whose last two recorded blood pressure measurements were <130/80 mmHg	Patients with a true risk score indicating a very high risk (SCORE ≥15%) or DM and age over 40 whose last two documented blood pressure readings were <130/80	Patients with a true risk score indicating a very high risk (SCORE ≥15%) or DM and age over 40
Is the blood pressure of lower risk patients controlled?	Proportion of lower risk patients (SCORE<15%) whose last two recorded blood pressure measurements were <140/90 mmHg	Patients with a true risk score indicating <15% whose last two documented blood pressure readings were <140/90	Patients with a true risk score indicating <15%
Are patients with existing CVD prescribed basic medications to reduce risk?	Proportion of patients with existing CVD prescribed a statin and aspirin and blood pressure lowering treatment	Patients with existing CVD prescribed a statin and aspirin and blood pressure lowering treatment	Patients with existing CVD
Is the blood glucose of diabetic patients controlled?	Proportion of diabetic patients with glycaemic control as defined by last two HbA1c measurements	Patients with diabetes 2 whose last two HbA1c measurements were below personal target as defined by MDA adapted WHO PEN 1	Patients with diabetes type 2
Is the blood pressure of hypertensive patients controlled?	Proportion of confirmed hypertensive patients whose SBP is <140/90 at last two visits	Patients with confirmed hypertension whose last two blood pressure readings were <140/90	Patients with confirmed hypertension
What is the prevalence of high blood pressure?	Proportion of people whose last two systolic blood pressure reading are 140 mmHg or above	Patients whose last two systolic blood pressure readings were ≥140	All patients over 18

For peer review only

447 **Table 2.** Standardized data collection form used to extract data from individual patient records
448

**Data Collection Question**	**Answer**
What is your name? (Name of person extracting data)	
Date of Data Extraction (MM-DD-YYYY)	
Write the Clinic Name	
Is this a duplicate extraction?	
If it is a duplicate extraction, enter the number you and your extraction partner have assigned to this file.	
Date of Birth (MM-DD-YYYY)	
Sex (M/F)	
Smoking Status (Y/M)	
Diagnosis of Hypertension (Y/N)	
Date of Hypertension Diagnosis (MM-DD-YYYY)	
Can you find one or more blood pressure readings? (Y/N)	
Most Recent Systolic Blood Pressure	
Most Recent Diastolic Blood Pressure	
Date of the Most Recent Blood Pressure Measurement (MM-DD-YYYY)	
Can you find a second most recent blood pressure reading? (Y/N)	
Second most recent systolic blood pressure	
Second most recent diastolic blood pressure	
Date of the second most recent systolic blood pressure (MM-DD-YYYY)	
Diagnosis of Diabetes (Type 1, Type 2, No)	
Can you find one or more HbA1c measurements? (Y/N)	
Most recent HbA1c reading (mmol/mol)	
Date of the most recent HbA1c measurement? (MM-DD-YYYY)	
Can you find another HbA1c measurement? (Y/N)	
Second most recent HbA1c reading (mmol/mol, otherwise specify unit)	
Date of the second most recent HbA1c reading? (MM-DD-YYYY)	
Can you find one or more total cholesterol measurements? (Y/N)	
Most recent total cholesterol reading (mmol/L)	
Date of the most recent cholesterol reading (MM-DD-YYYY)	
Can you find another cholesterol measurement? (Y/N)	
Second most recent cholesterol reading (mmol/L)	
Date of the second most recent cholesterol reading (MM-DD-YYYY)	
Was the patient prescribed a statin? (Y/N)	
What was the date of the statin prescription? (MM-DD-YYYY)	

For peer review only

What was the drug and dose?	
Does the patient have existing CVD? (Y/N)	
State the type of CVD	
Has the patient been prescribed acetylsalicylic acid (ASA or aspirin)? (Y/N)	
What was the most recent date that ASA was prescribed? (MM-DD-YYYY)	
Has the patient been prescribed anti-hypertensives? (Y/N)	
What was the most recent date that anti-hypertensives were prescribed? (MM-DD-YYYY)	
Can you find a documented ESC SCORE risk score? (Y/N)	
Enter the most recent documented ESC SCORE risk score (%)	
What was the date the risk score was documented? (MM-DD-YYYY)	
Please record any important notes about the data extraction here. Examples include an error you think may have been made, clarification of the units for measurements (e.g. mmol/L vs mg/dL). Or notes that you would like for yourself.	

449

For peer review only

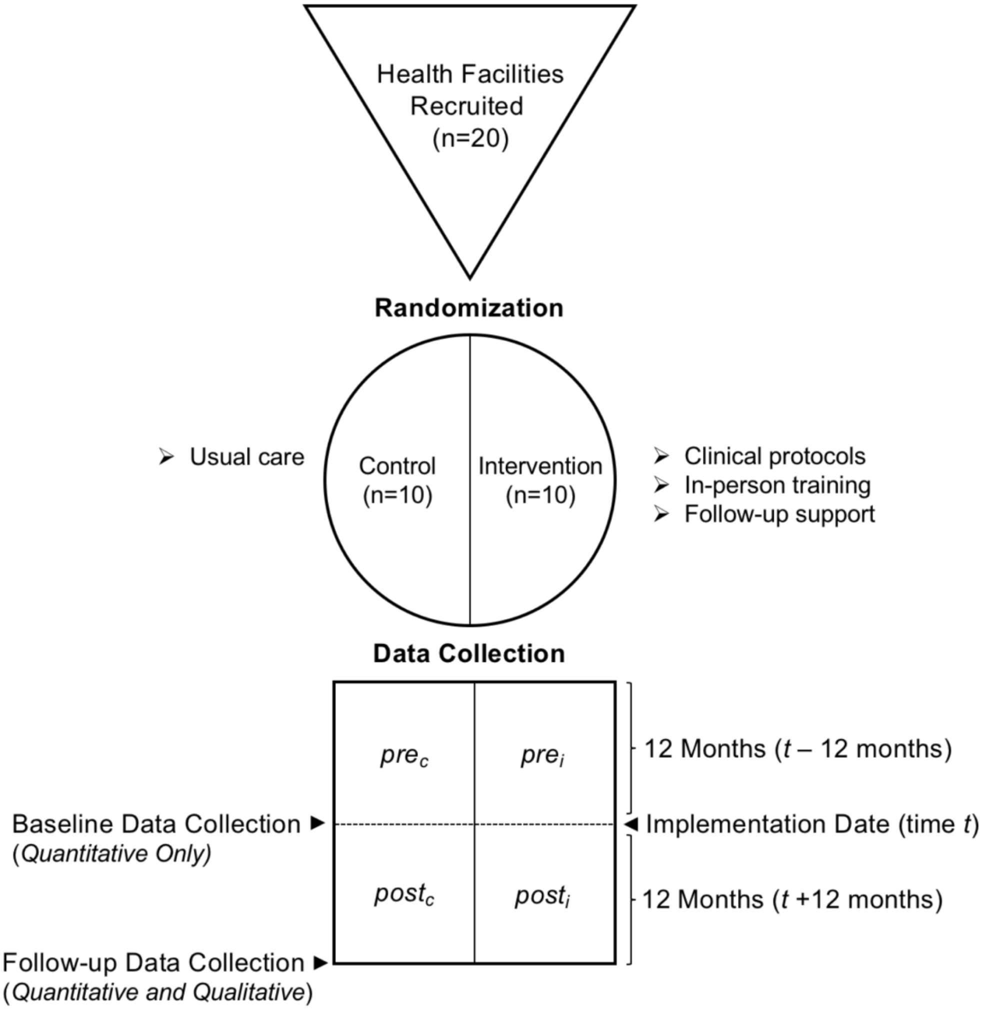

Figure 1. Illustration using the GATE frame structure (14) of the mixed methods evaluation design

184x193mm (300 x 300 DPI)
